# A scoping review of COVID-19 vaccine hesitancy: refusal rate, associated factors, and strategies to reduce

**DOI:** 10.3389/fpubh.2024.1382849

**Published:** 2024-10-15

**Authors:** Rona Bahreini, Mehran Sardareh, Morteza Arab-Zozani

**Affiliations:** ^1^Iranian Center of Excellence in Health Management (IceHM), Tabriz University of Medical Sciences, Tabriz, Iran; ^2^Student Research Committee, Tabriz University of Medical Sciences, Tabriz, Iran; ^3^Social Determinants of Health Research Center, Birjand University of Medical Sciences, Birjand, Iran

**Keywords:** COVID-19, vaccine hesitancy, refusal rate, strategy, scoping review

## Abstract

**Objective:**

This study aimed to investigate the evidence regarding vaccine hesitancy including refusal rate, associated factors, and potential strategies to reduce it.

**Methods:**

This is a scoping review. Three main databases such as PubMed, Scopus, and Web of Science were searched from 1 January 2020 to 1 January 2023. All original studies in the English language that investigated one of our domains (vaccine hesitancy rate, factors associated with vaccine hesitancy, and the ways/interventions to overcome or decrease vaccine hesitancy) among the general population were included in this study. The data were charted using tables and figures. In addition, a content analysis was conducted using the 3C model of vaccine hesitancy (Confidence, Complacency, and Convenience) that was previously introduced by the WHO.

**Results:**

Finally, 184 studies were included in this review. Of these, 165, 181, and 124 studies reported the vaccine hesitancy rate, associated factors, and interventions to reduce or overcome vaccine hesitancy, respectively. Factors affecting the hesitancy rate were categorized into 4 themes and 18 sub-themes (contextual factors, confidence barriers, complacency barriers, and convenience barriers).

**Conclusion:**

Vaccine hesitancy (VH) rate and the factors affecting it are different according to different populations, contexts, and data collection tools that need to be investigated in specific populations and contexts. The need to conduct studies at the national and international levels regarding the reasons for vaccine refusal, the factors affecting it, and ways to deal with it still remains. Designing a comprehensive tool will facilitate comparisons between different populations and different locations.

## Introduction

The World Health Organization (WHO) declared coronavirus disease 2019 (COVID-19) a global pandemic in March 2020 (1, 2). The COVID-19 pandemic has caused adverse health and socioeconomic impacts; as of January 2024, approximately 6.5 million people have died around the world (3). In May 2020, people all over the world agreed that getting vaccinated was important to stop the spread of COVID-19 (2, 4). The quick development of a COVID-19 vaccine gave hope for life to go back to normal. Despite the successful public vaccine uptake, many people delayed or refused to take the vaccination (5, 6).

With the development of multiple vaccines, there have been discussions about vaccine hesitancy (7). Although the effectiveness of vaccines depends on their availability, the increasing availability of vaccines has created the problem of VH. Vaccine hesitancy, which ranges from uncertainty about vaccines to outright opposition, was identified by the WHO in 2019 as one of the most important health problems in the world. This hesitancy poses challenges in controlling diseases that vaccines can prevent (8, 9).

Vaccine hesitancy is defined by the WHO as “the delay in the acceptance or refusal to vaccinate despite the availability of vaccine services” (10). Vaccine hesitancy is a complex phenomenon that is influenced by a wide range of contextual, individual, and group factors, including geographical, cultural, and socio-demographic factors, socioeconomic status, and perceptions of risk (1, 11, 12). Several studies explored COVID-19 vaccine acceptance and its determinants in China, Indonesia, Italy, Ireland, Japan, the United Kingdom, and the United States between March and December 2020 (7). Some of these studies indicated that factors such as age, income, and education levels are associated with a higher likelihood of accepting a vaccine (13–15).

A survey found four important issues: (1) People with chronic diseases worry more about getting COVID-19. (2) People wonder how being vaccinated will affect their chronic disease. (3) Some people are not sure if the COVID-19 vaccine is helpful. (4) There is too much information about COVID-19, and it confuses and worries patients (16).

Several reasons including personal and social are associated with vaccine hesitancy among parents. Gender, nationality, occupation, and being a healthcare worker were factors affecting the vaccination acceptance participants (17). More people were willing to get vaccinated if they trusted the government, had gotten a flu shot before, and saw COVID-19 as a danger to themselves and their community (15, 18–20).

Understanding the factors affecting vaccine hesitancy and identifying effective interventions can help with COVID-19 vaccination coverage and improve the healthcare system’s preparedness to plan and implement policies aimed at improving prevention programs for vaccine-preventable diseases. Many cross-sectional and review studies have been conducted in this field. However, there are few studies that report and synthesize the vaccine hesitancy rate, factors associated with vaccine refusal, and interventions to overcome or decrease vaccine hesitancy. This scoping review aimed to identify and synthesize the available evidence in the three dimensions of vaccine hesitancy rate, effective factors, and interventions to prevent it.

## Subjects and methods

### Protocol and registration

This scoping review was conducted based on the Joanna Briggs Institute guidelines (21). Furthermore, we reported this study following the Preferred Reporting Items for Systematic Reviews and Meta-Analyses extension for Scoping Reviews (PRISMA-ScR) (22).

### Eligibility criteria

All original studies that investigated at least one of our domains of vaccine hesitancy or refusal rate, factors associated with hesitancy or refusal of the vaccine, and the ways of interventions to overcome or decrease this phenomenon among the general population were included in this study. All non-English language studies were excluded from the study. We also excluded the studies if the full text was not available through database search or contact with authors.

### Information sources and search

Our search was conducted from 1 January 2020 to 1 January 2023 in three electronic databases: PubMed, Scopus, and Web of Science (WoS), using a combination of MeSH terms and free terms. Our general keywords were as follows: COVID-19, vaccine, and hesitancy. Full search strategies for databases are available in Supplementary material S1. We also searched the references of the included studies.

### Selection of sources of evidence

All records were imported to EndNote software version 20, and duplicates were removed. Two independent reviewers piloted screening with a random sample of 10 studies based on eligibility criteria (agreement rate: 93%), and disagreements were resolved with a third reviewer. Then, they started screening based on title, abstract, and full text. The final included studies were entered into the charting process.

### Data charting process and data items

To increase the agreement between reviewers, a data charting form was developed and independently piloted on a random sample of 10 included articles. This form includes data items of the first author, corresponding author, publication year, study design, country of origin, data collection period, participant group, sample size, ethical approval, funding statement, mean age, gender percent, the objective of the study, vaccine hesitancy or refusal rate, factors associated with or affecting hesitancy rate, and interventions to reduce or overcome hesitancy rate. Our definition of hesitancy rate was based on the WHO definition: “delay in acceptance or refusal of vaccines despite availability of vaccination services” (23).

### Synthesis of results

We categorized included studies by whether the major focus was vaccine hesitancy. The main domains were vaccine hesitancy rate, factors associated with vaccine hesitancy, and interventions to reduce or overcome hesitancy. The data were charted using tables and figures. Furthermore, we performed a content analysis using the 3C (Confidence, Complacency, and Convenience) model of vaccine hesitancy that was previously introduced by the WHO for categorizing the factors associated with vaccine hesitancy (24).

### Patient and public involvement

As our design is a scoping review, patients or the public were not involved in any stage of the study including design, data collection, synthesis, reporting, and dissemination of this research.

## Results

### Selection of sources of evidence

**Figure 1:**
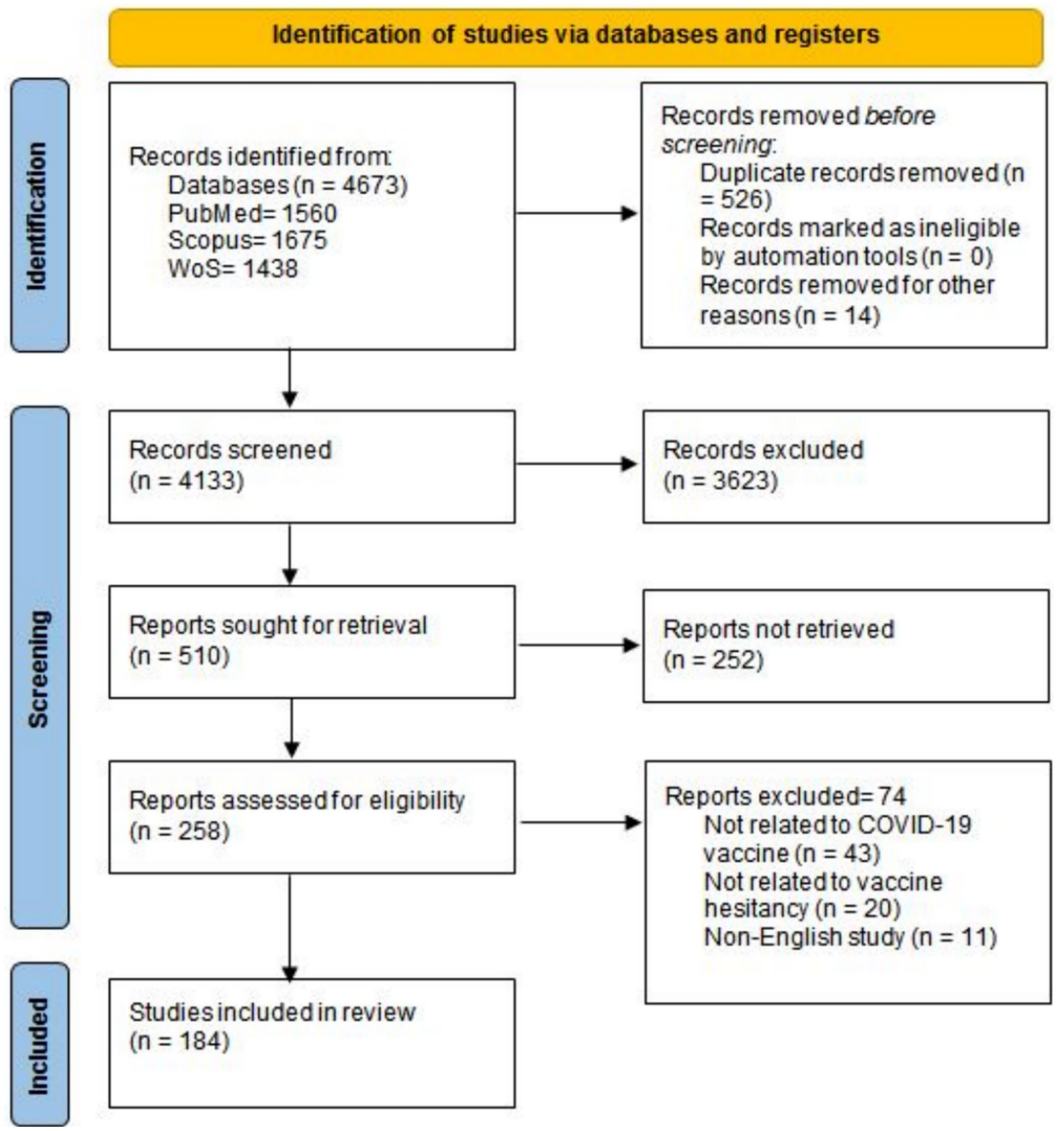
PRISMA flow diagram.

Our database search resulted in 4673 records. After duplication and initial screening, 258 records met the eligibility criteria and were considered for full-text screening. Finally, 184 articles were included in this scoping review (5, 7, 12, 25–197). The PRISMA flow diagram of the study selection process is represented in Figure 1.

### Characteristics of sources of evidence

**Figure 2:**
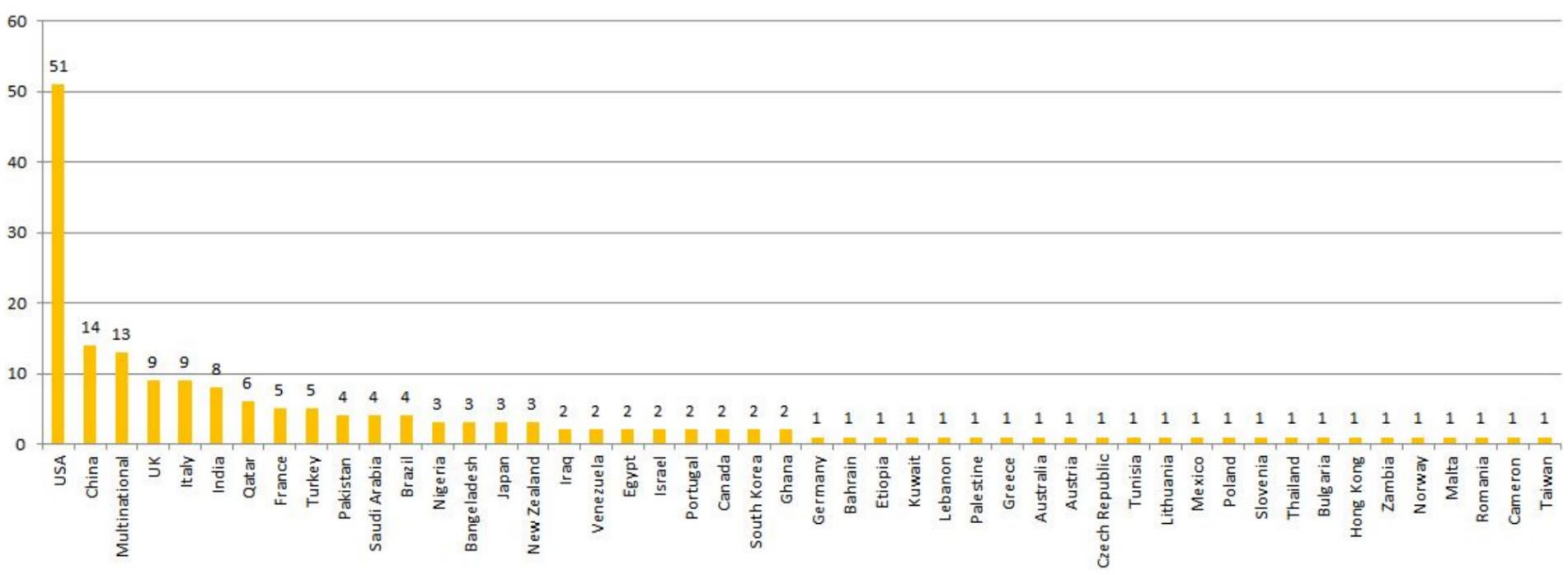
Distribution of the final included studies by country.

Studies were conducted across five continents: 60 from the Americas, 58 from Asia, 38 from Europe, 11 from Africa, and 4 from Oceania. Thirteen studies were multinational, 51 studies (27.7%) were conducted in the USA, and 14 studies were conducted in China (7.6%). The distribution of the included studies between countries is represented in Figure 2.

Of the 184 included studies, 175 were cross-sectional, 4 were cohort, 3 were qualitative, and 2 were randomized clinical trials (RCTs). The highest number of articles published in 2021 was 179. Funding and ethical statements were reported in 144 and 137 studies, respectively. The sample size of the included studies ranged from 20 to more than 1 million. Of the 184 included studies, 165 (89.6%) reported the hesitancy rate regarding COVID-19 vaccines, 181 (98.3%) reported the factors associated with COVID-19 vaccine hesitancy, and 124 (67.4%) reported potential interventions to reduce or overcome COVID-19 vaccine hesitancy among participants. The summary characteristics of the included studies are reported in Table 1.

**Table 1:** Summary characteristics of the included studies (*n* = 184).

Characteristics	Number (*n* = 184)	Percentage (%)
Publication year		
2020	5	2.71
2021	179	97.3
Study design		
Cross-sectional	175	95.1
Cohort	4	2.2
RCTs	2	1.1
Qualitative	3	1.63
Continent		
Africa	11	5.97
America	60	32.6
Asia	58	31.5
Europe	38	20.6
Oceania	4	2.17
Multinational	13	7.06
Ethical statement		
Yes	137	74.4
No	47	25.6
Funding statement		
Yes	144	78.3
No	40	21.7
Sample size		
≤200	14	7.6
201–500	44	23.9
501–1,000	29	15.8
>1,000	97	52.7
Domain		
Hesitancy rate	165	89.6
Factors	181	98.3
Interventions	124	67.4

The hesitancy rate of the vaccine was different among the studies depending on the type of population, sample size, and method of conducting the study, and it ranged from less than 3% to more than 80%. Factors affecting vaccine hesitancy and interventions suggested by individual studies to overcome it are reported in Supplementary materials S2, S3.

### Synthesis of results

Factors affecting the hesitancy rate are categorized into 4 themes and 18 sub-themes including contextual factors, confidence barriers, complacency barriers, and convenience barriers. Contextual factors include two main sub-themes: socio-demographic predictors and political-economic-cultural factors. Confidence barriers include seven main sub-themes: vaccine effectiveness and efficiency; fear and concerns about vaccine development; fear and concerns about side effects, adverse events, and death after vaccination; reliability and trustworthiness of received information from the vaccine program; confidence in safety; trust in authorities, providers, and institutions; and trust in science. Complacency barriers include two main sub-themes: beliefs about identifying the severity and risk of COVID-19 and preference for alternative immunity methods. Convenience barriers to getting vaccinated include being able to physically go and get vaccinated, being able to afford it, living in an area where it is available, understanding health information, having access to good vaccination services, finding the time and place to get vaccinated, and considering cultural factors. The overall themes, sub-themes, and code summary are reported in Table 2.

**Table 2:** Factor affecting the hesitancy rate.

Main theme	Sub-theme
Contextual factors	Socio-demographic predictors
	Political-economic-cultural factors
Confidence barriers	Vaccine effectiveness and efficiency
	Fear and concerns about vaccine development
	Fear and concerns about side effects, adverse events, and death after vaccination
	The dependability and credibility of the information obtained from the vaccine program
	Confidence in safety
	Trust in authorities, providers, and institutions
	Trust in science
Complacency barriers	Beliefs about identifying the severity and risk of COVID-19
	Preference for alternative immunity methods
Convenience barriers	Physically capable of receiving the vaccine
	Able to afford the cost of vaccination
	Reside in an area and community where receiving a vaccination is accessible
	Health and language literacy
	Standard of vaccination service
	The designated schedule and location for receiving the vaccination
	The cultural background of the group

## Discussion

Overall, 184 articles were included in this scoping review. These articles report at least one of our domains investigated in this review including hesitancy rate, factor effecting, and strategy to prevent or reduce hesitancy regarding COVID-19 vaccines. Of the 184 included studies, 165 (89.6%) reported the hesitancy rate regarding COVID-19 vaccines, 181 (98.3%) reported the factors associated with COVID-19 vaccine hesitancy, and 124 (67.4%) reported potential interventions to reduce or overcome COVID-19 vaccine hesitancy among participants. COVID-19 vaccine hesitancy varied significantly based on the population, sample size, and method of conducting the studies, with reported rates ranging from less than 3% to more than 80% in the included studies. In addition, the main factors affecting the hesitancy rate were categorized into four groups: contextual factors, confidence barriers, complacency barriers, and convenience barriers. Public education campaigns, consultation programs, patient engagement, providing detailed information and shared decision-making programs, streaming educational content through media and broadcast channels, strengthening positive attitudes to vaccination, and reducing conspiracy suspicions were among the important interventions to reduce vaccine hesitancy during the COVID-19 pandemic.

### Vaccine hesitancy rate

The rate of vaccine hesitancy was different among the studies depending on the type of population, sample size, and method of conducting the study, and it ranged from less than 3% to more than 80%. The findings of several systematic and scoping reviews are consistent with our results.

Our results were comparable to those of a scoping review conducted by Ackah et al. on an African population. Based on their results, the vaccine acceptance rate ranged from 6.9 to 97.9% (198). Another systematic review conducted by Sallam showed similar results, indicating that the highest COVID-19 vaccine acceptance rate was 97% in Ecuador, while the lowest was 23.6% in Kuwait (199).

The results of a scoping review that was conducted in high-income countries also showed a similar result to our study. Based on this scoping review, the rates of vaccine hesitancy across high-income countries or regions ranged from 7 to 77.9% (200). Furthermore, the results of a worldwide scoping review of COVID-19 vaccine hesitancy showed that people in different countries had varying percentages of vaccine uptake (28–86.1%), vaccine hesitancy (10–57.8%), and vaccine refusal (0–24%) (201).

### Factor associated

In various studies, several factors have been mentioned that are related to not accepting the vaccine or accepting the vaccine in the case of COVID-19. These influencing factors have been different depending on the geographical context; the participants; demographic characteristics of the population; background factors; and other cultural, social, economic, and political factors in the studies. However, many of these factors have been similar in different studies, and even their causal relationships have been investigated.

A study was carried out to explore the level and factors contributing to COVID-19 vaccine hesitancy in South Africa in order to guide the development of interventions to combat it. The study found that COVID-19 vaccine hesitancy in South Africa is primarily influenced by social factors such as age, race, education, politics, location, and employment status (202). Another study identified socio-demographic factors as the individual determinants of vaccine hesitancy. Right-wing political affiliation was the main socio-demographic psychological determinant (89). Political affiliation was identified as one of the contextual factors in our findings.

A narrative review was conducted to explore and examine the issue of vaccine hesitancy amid the COVID-19 pandemic. The review identified various factors that impact individuals’ decisions to accept or reject vaccines, such as ethnicity, employment status, religious beliefs, political views, gender, age, education level, income, and other factors (203).

A scoping review was carried out on 60 studies from around the world to explore vaccine hesitancy and acceptance. Through qualitative analysis, this study identified factors that influence people’s decisions on vaccination in various cultural and demographic settings. These factors include risk perceptions, trust in healthcare systems, solidarity, past experiences with vaccines, misinformation, concerns about vaccine side effects, and political beliefs, all of which play a role in addressing or reducing vaccine hesitancy (204).

The WHO acknowledges that vaccine hesitancy poses a significant risk to public health. A recent study investigated how attitudes, norms, and perceived behavioral control influence vaccination intentions. The study found that these factors were important predictors of vaccination intentions, with attitude being the most influential (205). In our study, a positive attitude toward the government has been mentioned as an effective factor in vaccine hesitancy.

Vaccine hesitancy, characterized by a lack of trust in vaccinations and/or indifference toward them, can result in postponing or rejecting vaccination even when it is accessible. This poses a significant risk to the effectiveness of COVID-19 vaccination initiatives. Factors such as the quick development of vaccines, misinformation spread through mainstream and social media, the divided socio-political climate, and the challenges of implementing widespread vaccination campaigns may erode confidence in vaccination and heighten apathy toward COVID-19 vaccination efforts (206).

Various factors that impact the decision-making process regarding vaccines are diverse, intricate, and dependent on the specific context. These factors can be triggered or exacerbated by unregulated online information or misinformation. Kassianos et al. (207) addressed the most common concerns regarding the COVID-19 vaccination. The rise of new variants of COVID-19 has contributed to vaccine hesitancy. Healthcare professionals, who are considered reliable sources of information, need to be provided with sufficient resources and practical advice to help them address concerns effectively (207). Advice and recommendations from health professionals about getting or not getting vaccinated were discussed as one of the effective factors in vaccine hesitancy.

Although vaccination has proven to be an effective tool in combating the worldwide COVID-19 pandemic, vaccine hesitancy has become a significant challenge in various countries, including Africa. A scoping review was conducted to consolidate the current research on vaccine hesitancy in Africa. The primary reasons for hesitancy included worries about vaccine safety and potential side effects, distrust in pharmaceutical companies, and exposure to misinformation or contradictory information from the media. Factors linked to a more favorable view of vaccines included being male, having a higher education level, and a fear of contracting the virus (198). These reasons were also discussed in our findings.

According to the classification of the WHO model, health and language literacy is one of the convenience barriers, which includes negative stories, misinformation, and misperceptions focusing on the vaccine and personal knowledge. A study examined the frequency and reasons behind COVID-19 vaccine hesitancy among individuals with mental health conditions. Common factors contributing to hesitancy include distrust, false information, belief in conspiracy theories, and negative views on vaccines. Mental health disorders, particularly anxiety and phobias, may heighten the likelihood of vaccine hesitancy (208).

Vaccine hesitancy is a significant obstacle to the acceptance of the COVID-19 vaccine. A scoping review was conducted to summarize the rates of COVID-19 hesitancy and its determinants in affluent countries or regions. Factors such as younger age, gender, non-white ethnicity, and lower education levels were commonly linked to increased vaccine hesitancy. Other factors included not having a recent history of influenza vaccination, a lower perceived risk of contracting COVID-19, less fear of the virus, belief in the mildness of COVID-19, and absence of chronic medical conditions. Specific concerns about vaccine safety and effectiveness, as well as worries about the rapid development of COVID-19 vaccines, were also associated with increased vaccine hesitancy (200). All these factors are consistent with our findings.

### Strategies to overcome

Considering the wide range of factors affecting vaccine hesitancy, various studies have proposed various interventions to overcome this issue. These interventions have been at various levels, including media, service delivery, community education, and even decision-making processes.

One of the important interventions that has been mentioned in many studies is holding various campaigns to educate people at the community, individual, and family level (28, 66, 134). Some of these training campaigns have been focused on equipping service providers and health sector employees, empowering them to effectively transfer this knowledge to the general public (42). In some of these campaigns, the target population has included less privileged and less educated people because some studies have shown that the level of education is one of the factors affecting knowledge and attitude in this field (47). The way of conducting these campaigns has also been different. In some cases, campaigns have been organized at the community level, following the principles of prevention. Some other campaigns have been held online through media due to the COVID-19 situation (32, 171).

Another recommended intervention in this field has been holding counseling sessions and establishing hotlines to communicate with consultants in the field of public health. In some societies, this disease is considered a kind of stigma, making online communication and telephone consultations very welcome due to the confidentiality and anonymity they offer. On the other hand, some obstacles that exist in face-to-face communication, such as the shyness of raising the issue, are reduced in such a mode of communication, and people raise relevant issues more easily (88, 186, 209).

Another important intervention for reducing vaccine hesitancy is holding educational workshops to explain the advantages and disadvantages of vaccination for different target populations. By holding such workshops and training programs, while increasing people’s awareness, people are given the opportunity to know the advantages and disadvantages of an intervention and decide whether or not to receive it. In this regard, communication between doctors and patients is crucial, as because healthcare professionals are important sources for increasing confidence in vaccination. Patients tend to trust these individuals more, which facilitates acceptance of their recommendations (81, 114, 174).

Some studies showed that recommending vaccination and presenting its advantages and disadvantages through celebrities can have good effects on the general population due to their fame and followers (91). Furthermore, advertising during major events featuring celebrities can also help in this area. However, due to the conditions surrounding this disease and the need to maintain preventive measures, many of these events were held in closed settings at that time, which may have reduced their effectiveness (60, 149).

## Conclusion

Based on our study, the vaccine hesitancy rate and the factors affecting it vary across different populations, contexts, and data collection methods. This underscores the need for further investigation in specific populations and contexts. The need to conduct studies at the national and international levels regarding the reasons for vaccine refusal, the factors affecting it, and ways to deal with it is still pending. Designing a comprehensive tool will facilitate comparisons between different populations and different locations. Health and medicine authorities should strengthen the dissemination of vaccination-related knowledge for patients such as an expert consensus or guidelines through various media. Some key points should be emphasized in the knowledge sharing about vaccination, including the importance of vaccination, the safety and side effects of COVID-19 vaccines, and predictions regarding epidemiological trends of COVID-19.

## Data Availability

The original contributions presented in the study are included in the article/Supplementary material, further inquiries can be directed to the corresponding author.
