# Supplementary material for: A scoping review of COVID-19 vaccine hesitancy: refusal rate, associated factors, and strategies to reduce
Source: Front Public Health. 2024 Oct 15;12:1382849. doi: 10.3389/fpubh.2024.1382849 (PMC11518786; doi:10.3389/fpubh.2024.1382849)
Supplement: Supplementary file 1 [file Table_1.DOC]

**A Scoping Review of COVID-19 Vaccine Hesitancy; Refusal Rate, Factor Associated and Strategies to Reduce or Overcome**

**Journal of Public Health**

**Online Resource 1** Detailed search strategies

| **Database** | **Query** | **Results** |
| --- | --- | --- |
| PubMed | ("COVID-19 vaccine hesitancy"[All Fields] OR ("vaccine hesitancy"[All Fields] AND ("covid 19"[All Fields] OR "covid 19"[MeSH Terms] OR "covid 19 vaccines"[All Fields] OR "covid 19 vaccines"[MeSH Terms] OR "covid 19 serotherapy"[All Fields] OR "covid 19 nucleic acid testing"[All Fields] OR "covid 19 nucleic acid testing"[MeSH Terms] OR "covid 19 serological testing"[All Fields] OR "covid 19 serological testing"[MeSH Terms] OR "covid 19 testing"[All Fields] OR "covid 19 testing"[MeSH Terms] OR "sars cov 2"[All Fields] OR "sars cov 2"[MeSH Terms] OR "severe acute respiratory syndrome coronavirus 2"[All Fields] OR "ncov"[All Fields] OR "2019 ncov"[All Fields] OR (("coronavirus"[MeSH Terms] OR "coronavirus"[All Fields] OR "cov"[All Fields]) AND 2019/11/01:3000/12/31[Date - Publication]))) OR (("covid 19"[All Fields] OR "covid 19"[MeSH Terms] OR "covid 19 vaccines"[All Fields] OR "covid 19 vaccines"[MeSH Terms] OR "covid 19 serotherapy"[All Fields] OR "covid 19 nucleic acid testing"[All Fields] OR "covid 19 nucleic acid testing"[MeSH Terms] OR "covid 19 serological testing"[All Fields] OR "covid 19 serological testing"[MeSH Terms] OR "covid 19 testing"[All Fields] OR "covid 19 testing"[MeSH Terms] OR "sars cov 2"[All Fields] OR "sars cov 2"[MeSH Terms] OR "severe acute respiratory syndrome coronavirus 2"[All Fields] OR "ncov"[All Fields] OR "2019 ncov"[All Fields] OR (("coronavirus"[MeSH Terms] OR "coronavirus"[All Fields] OR "cov"[All Fields]) AND 2019/11/01:3000/12/31[Date - Publication])) AND ("vaccin"[Supplementary Concept] OR "vaccin"[All Fields] OR "vaccination"[MeSH Terms] OR "vaccination"[All Fields] OR "vaccinable"[All Fields] OR "vaccinal"[All Fields] OR "vaccinate"[All Fields] OR "vaccinated"[All Fields] OR "vaccinates"[All Fields] OR "vaccinating"[All Fields] OR "vaccinations"[All Fields] OR "vaccination s"[All Fields] OR "vaccinator"[All Fields] OR "vaccinators"[All Fields] OR "vaccine s"[All Fields] OR "vaccined"[All Fields] OR "vaccines"[MeSH Terms] OR "vaccines"[All Fields] OR "vaccine"[All Fields] OR "vaccins"[All Fields]) AND ("hesitance"[All Fields] OR "hesitancies"[All Fields] OR "hesitancy"[All Fields] OR "hesitant"[All Fields] OR "hesitate"[All Fields] OR "hesitated"[All Fields] OR "hesitating"[All Fields] OR "hesitation"[All Fields] OR "hesitations"[All Fields]))) AND (2020:2021[pdat]) | 1560 |
| Scopus | ( TITLE-ABS-KEY ( "COVID-19 vaccine hesitancy" )  OR  TITLE-ABS-KEY ( "vaccine hesitancy"  AND  covid-19 )  OR  TITLE-ABS-KEY ( covid-19  AND  vaccine  AND  hesitancy ) )  AND  ( LIMIT-TO ( PUBYEAR ,  2021 )  OR  LIMIT-TO ( PUBYEAR ,  2020 ) ) | 1675 |
| WoS | “vaccine hesitancy” (Topic)  COVID-19 (Topic)  vaccine (Topic)  hesitancy (Topic)  #1 AND #2  #2 AND #3 AND #4  “COVID-19 vaccine hesitancy” (Topic)  #5 OR #6 OR #7  #5 OR #6 OR #7 and 2020 or 2021 (Publication Years) | 1438 |
