# Supplementary material for: A scoping review of COVID-19 vaccine hesitancy: refusal rate, associated factors, and strategies to reduce
Source: Front Public Health. 2024 Oct 15;12:1382849. doi: 10.3389/fpubh.2024.1382849 (PMC11518786; doi:10.3389/fpubh.2024.1382849)
Supplement: Supplementary file 2 [file Table_2.DOC]

**A Scoping Review of COVID-19 Vaccine Hesitancy; Refusal Rate, Factor Associated and Strategies to Reduce or Overcome**

**Journal of Public Health**

**Online Resource 2** Factors affecting vaccine hesitancy by individual studies

| Authors/Year | Factors |
| --- | --- |
| Lu et al, 2021 [1] | **related to**: own vaccination against COVID-19, children’s uptake of existing vaccines and parental acceptance of childhood COVID-19 vaccines/Chinese; poor educational level; age; parental attitudes on childhood COVID-19 vaccines/**reasons:** vaccines are [not] important for myself; vaccines are [not] effective; getting vaccinated id [not] important for others; all vaccines offered by the government are [not] beneficial; the information i received about vaccines is [not] trustworthy; getting vaccines is [not] a good way to protect myself; generally i [don't] do what my doctor recommends about vaccines; new vaccines carry more risks than older vaccines; i am concerned about serious adverse effects of vaccines; i do not need for diseases not common. |
| Abdel-Raheem and Alkhammash, R. 2021 [2] | readers of the news articles, but not of the cartoons, are susceptible to framing effects |
| Abdulah, 2021 [3] | Importance of COVID‐19 vaccine health; Effectiveness of COVID‐19 vaccines; Importance of any vaccinated person for the health of others; Beneficial of all COVID‐19 vaccines; More risks of new vaccines versus older vaccines; Reliability and trustworthiness of received information from the vaccine program; Receiving a COVID‐19 vaccine as a good way of protection against the disease; Following the instructions of the doctor or health care provider; Concerns about the serious adverse effects of the COVID‐19 vaccine; Not required to get a COVID‐19 vaccine due to begin common the disease./**Contributing factors:** level of education; Occupation setting; Concerns of adverse side‐effects; Importance of COVID‐19 vaccine health; Effectiveness of COVID‐19 vaccines; Importance of any vaccinated person for the health of others; Beneficial of all COVID‐19 vaccines; More risks of new vaccines versus older vaccines; Reliability and trustworthiness of received information from the vaccine; Receiving a COVID‐19 vaccine as a good way of protection against the; Following the instructions of the doctor or health care provider; Concerns about the serious adverse effects of the COVID‐19 vaccine; Not required to get a COVID‐19 vaccine due to begin common the disease; infection by the COVID‐19. |
| Abouhala, et al. 2021 [4] | **associated with vaccine intention:** age, gender, and religiosity |
| Acheampong, et al.2021 [5] | gender, age, educational attainment, and region of residence/**reasons for taking vaccines:** it will help me protect family, friends, and other people in the community; the vaccine is effective at preventing me from getting COVID-19; and I have a public health responsibility to help fight the pandemic/**reasons undecided about taking vaccines:** not being well informed about the possible effects of the vaccine; not being sure that the vaccine is clinically safe; and not being sure that the vaccine is effective to prevent them from getting COVID-19/**Reasons for not wanting to take the vaccine:** I am not sure that the vaccine is clinically safe; I am not well informed about the possible effects of the vaccine; and not being sure that the vaccine is effective to prevent them from getting COVID-19/**the decision to take, remain undecided, or unlikely to take the COVID-19 vaccine is significantly influenced by:** age, gender, and primary source of information |
| Adigwe, . 2021 [6] | **reasons:** I do not believe COVID-19 exist; I am against vaccine in general; the risk of getting COVID-19 is low; I am not against taking vaccine; worried about the side effect |
| Aemro, et al. 2021 [7] | **predictors:** younger age, non-compliance with COVID-19 infection preventive measures, hearing unclear COVID-19-related information from health authorities, considering oneself at low risk of getting COVID-19 infection and not being sure regarding tolerable side effects of the vaccine |
| Alabdulla, et al. 2021 [8] | **more likely to be vaccine hesitators:** older, native Qataris, self-employed or retired, single, and female/**Worries around COVID-19 infection and its vaccine:** Worries about getting infected; Worries about a family member getting infected; Financial worries; Job-related worries; Worries of unavailability of a vaccine./ **associated with:** the belief that there has been insufficient testing of COVID-19 vaccines; the view that authorities are motivated by financial gain rather than health of people; natural exposure to germs and viruses gives the safest protection./variables associated with vaccination hesitancy: Age; Qatari Nationals; Female gender; Had the flu vaccine in the last 3 y; Worries that COVID-19 is a new disease and vaccines have not been fully tested; Feel safe after being vaccinated; Feel protected after getting vaccinated; Belief that natural exposure to germs and viruses give the safest protection |
| Aldakhil, et al. 2021 [9] | signiﬁcant association between vaccine hesitancy and low education levels/source of vaccine information/ **factors pertaining to lack of vaccine conﬁdence:** concerns about the vaccine’s safety and its side effects; vaccine was not effective; social inﬂuence/**predictor of mothers’ intentions to vaccinate their children against COVID-19:** vaccine hesitancy towards childhood immunization |
| Alfieri, et al. 2021 [10] | **Associations:** racial/ethnic groups, income, insurance type, and source of information |
| Ali, M and Hossain, A. 2021 [11] | **Hesitancy was high:** males, over age 60, unemployed, from low-income families, from central Bangladesh including Dhaka, living in rented houses, tobacco users, politically affiliated, participants who did not believe in the vaccine’s effectiveness for Bangladeshis and those who did not have any physical illnesses in the last year. *(sociodemographic characteristics, health, and behavior of participants such as gender, age, marital status, income, employment status, tobacco use, history of illness, place of residence, and political affiliation.)*/**significantly associated with (predictors):** transgender, married, tobacco users, those who did not get any physical illnesses in the last year, those with political affiliations with opposition parties, those who believed COVID-19 vaccines will not be effective for Bangladeshis, and those who were slightly concerned or not concerned at all about themselves or a family member getting infected with COVID-19 in the next one year/**contributing factors:** preexisting indecisiveness, cultural and religious views, lack of belief in the scientific enterprise of medicine and public health especially among the older population, and lower levels of awareness |
| Alibrahim, J and Awad, A. 2021 [12] | **Reasons for uncertainty or unwillingness to vaccinate:** I do not believe that COVID-19 is a serious infection that requires vaccination; COVID-19 infection is a conspiracy and I do not believe that it exists; I am concerned about the efficacy of the COVID-19 vaccine in preventing me from getting the infection; I am concerned about the possible side effects of the COVID-19 vaccine; I do not yet have adequate information about the vaccine to decide; The development of the COVID-19 vaccine was rushed, and it has not been thoroughly tested before approval; The COVID-19 vaccine has not yet been taken by many people in the public to know its side effects; Some of the physicians posted videos on social media advising against receiving the vaccine raising my suspicion; I have a strong immune system that protects me against the COVID-19 infection; I have allergies and am worried that I may have an allergic reaction to it./**Predictors:** age; material status; Residence area; Monthly income; non-smokers; those who are not feeling worried about catching a COVID-19 infection; those who do not know whether any of their family members have been infected with COVID-19 or died because of COVID-19 infection; those who do not have a large extent of confidence in Kuwait health system’s ability to handle the COVID-19 pandemic well; those who have not received influenza vaccine during the last year; those who refused or elected to forego a doctor-recommended vaccine for them or someone they are responsible for; those who did not receive adequate information from the public health authorities/their healthcare providers about the COVID-19 vaccines available in Kuwait; those who none of their first-degree family members received or are intending to receive the COVID-19 vaccine; and those who expressed high and intermediate levels of negative attitudes towards mistrust of vaccine benefit, concerns about commercial profiteering, preference for natural immunity, and the overall VAX score. |
| Allington, et al. 2021 [13] | associated with age, household income, level of education, level of coronavirus risk perception (possibly excluding perceived risk to people living in other countries), trust in government, scientific and medical authorities and informational reliance on legacy media./**predictors:** attitudes to vaccines in general and conspiracy suspicions with regards to coronavirus |
| Almaghaslah, et al.2021 [14] | **reasons:** I’m not sure of eligibility and registration process; I’m not sure of the effectiveness of the vaccination; I believe that natural immunity is sufﬁcient, and I don’t think I need to take the vaccine; I’ve heard on social media that the vaccine is not safe as it would contain the COVID-19 virus, so I’m worried about the side effects |
| Al-Mohaithef, et al. 2021 [15] | **factor affecting the decision in acceptance of the COVID-19 vaccine:** high-risk perception; higher trust in the healthcare system/**associated with intention to COVID-19 vaccination:** Marital status; Highest education; Family size |
| Al-Mulla, et al.2021 [16] | **Reasons given for unwillingness to take the COVID-19 vaccine:** vaccine is unsafe; vaccine is ineffective; against all vaccines; untrustworthy source of vaccine./**Determinants:** gender; age; Health-related discipline; Nationality; QU Status/**Other Determinants Inﬂuencing:** who considered it important to take the annual inﬂuenza vaccination; minimum length of time a testing process; participants’ worries about the rushed pace of development of a COVID-19 vaccine and the failure to detect potential side effects |
| Al-Wutayd, et al. 2021 [17] | **Sociodemographic Predictors:** ages (31 and 40 years); gender (females); Occupation (housewives); Education level (uneducated)./**Behavioral Predictors:** Urban residency; reservations about vaccine safety; uncertainty about its effectiveness; the failure of a vaccine to reduce hospitalization and death; unfelt need for vaccination awareness among public. /**Reasons:** Concerns about the vaccine’s side effects; Concerns on conspiracy theories about COVID-19 vaccine; Belief that the vaccine cannot save from COVID-19 infection; No need of vaccine because all the precautions are ensured (washing hands, wearing mask and gloves); No need of vaccine because of perceived protection of participant from COVID-19 due to their young age and good health; Fear of needles/syringes; Perceived acquisition of immunity against COVID-19 virus after contracting and recovering from COVID-19 infection (in past months); Wait and see the effects of vaccines in others/Insufficient information about vaccine./**willing to get vaccinated:** At least one chronic disease; knowing someone infected with COVID-19; trusting information from the ministry of health; and physicians. |
| Amuzie, . et al. 2021 [18] | **Sociodemographic predictors:** younger age; Marital status; Profession (doctor, nurse, other allied health professionals); lower income |
| Andrade, . 2021 [19] | there are statistically significant differences between ethnicities in scores regarding plans to get the vaccine, vaccine conspiracy beliefs, generic conspiracist beliefs, and perceived ethnic discrimination/ intention to get the vaccine by ethnicity with a statistically significant result regarding differences across ethnicities/marginalized ethnic groups in Venezuela are more prone to vaccine hesitancy, and also have greater levels of acceptance regarding conspiracy theories. Coefficients of correlation of ethnic discrimination with vaccine hesitancy and conspiracy beliefs are moderate. Coefficients of correlation of paranoid ideation with vaccine hesitancy and conspiracy beliefs are weak. |
| Andrade, . 2021 [20] | **predictive factor:** Ethnicity and religion/**factors:** low educational level/Income has a weak negative correlation |
| Asaoka, et al. 2021 [21] | **depressive symptoms** were worse among those who were unwilling ( poor mental health ) |
| Ashok et al. 2021 [22] | **association with vaccine acceptance:** Infected members in the immediate social network; COVID-19 knowledge; the safety of vaccines; and those who did not receive a flu vaccine last year |
| Badr, et al .2021 [23] | Work status; Marital status; Income; Social cognitive factors |
| Bagasra, et al. 2021 [24] | **predictor:** adults’ trust in information from the scientific community; less trust; racial differences; ethnic differences |
| Bagateli, et al.2021 [25] | **associated with refusal of the COVID-19 vaccine:** Number of Children in Household; Education; Household Income/**reasons:** concerned about serious side effects of the vaccines and concerns about their safety |
| Baniak, et al.2021 [26] | **Concerns:** its side effects, the lack of evidence on its effectiveness, and the potential for the vaccine to be ineffective in mitigating risk/**signiﬁcant variables of Vaccine Uptake:** adequate information about the expectations of the vaccine, adequate information to make an informed decision about whether to receive the vaccine or not, conﬁdence in safety, conﬁdence in effectiveness, expectations of effectiveness, and work experience/**Predictor of Vaccine Uptake:** Conﬁdence in Safety; Work Experience/**hesitant group reported:** not having inadequate information to make an informed decision about whether to receive the vaccine and about vaccine expectations |
| Belingheri, et al. 2021 [27] | **the intention to adhere to the COVID-19 vaccination program was associated:** Age > 55 years; Flu vaccination season 2020–21; Flu vaccination in previous seasons; COVID-19 diagnosis (personal)/reason for opposing vaccination: lack of information about COVID-19 vaccine; COVID-19 vaccine is unsafe;; fear of adverse events; pharmaceutical companies influence decisions about vaccination policies; previous diagnosis of COVID-19; sub-optimal protective efficacy; disagree to vaccination; COVID-19 is not a threatening disease./**reasons for supporting vaccination:** to protect myself; to protect patients; to return to normal activities; to not miss days of work; to comply with health ministry recommendations; to not wear masks anymore |
| Bendau, et al. 2021 [28] | COVID-19-related anxiety, and fears of infection and health-related consequences correlated significantly positively with vaccine acceptance/, social and economic fears showed significant negative associations with vaccination willingness. |
| Blanchi, et al. 2021 [29] | **associated with:** younger age, lower perception of disease severity, vaccine efficacy, lower trust in vaccination, in the health care system and scientists, concerns about side effects, living in France, |
| Bogart, et al. 2021 [30] | general COVID-19 mistrust |
| Botwe, et al. 2021 [31] | **reasons:** Not convinced about its effectiveness and side effects/Not interested./No evidence on effects on my type of race and population (Ghanaians)./Due to adverse reactions to previous vaccines eg. H1N1 vaccine in 2012./Lack of adequate information and education./Lack of enough research evidence to back the potency of the vaccines./Not sure if it affects fertility./I have heard of some severe side effects./I do not know whether people with underlying conditions can take it. |
| Bou Hamdan, et al.2021 [32] | **Vaccine hesitancy associated with:** nationality, residency status, university rank, agreement with conspiracies was observed; COVID-19 infection history among people in the same social circle; Hospitalization due to COVID-19 among people in the same social circle; Flu vaccination history in the past few years; Health behaviour during the past month; Social distancing of minimum 6 ft; Washing hands; Avoiding indoor spaces; Avoiding close contact with COVID-19 positive cases; Intentionally not going out; Views regarding COVID-19 vaccination; Mainstream media creating unnecessary fear about COVID-19; COVID-19 vaccine is not needed; Potential harm of COVID-19 infection is exaggerated; Death/year from flu are millions more than that of COVID-19; COVID-19 vaccine is more dangerous than the disease itself; Pharmaceutical companies have hidden information about the vaccine’s bad health outcomes; No sense to get the vaccine, higher power manipulates health outcomes; COVID-19 vaccine is an attempt to take away personal freedom; Government is using the vaccine to control population/**Factors associated with hesitancy:** Importance of decreasing odds of dying from COVID-19; Feelings towards getting the vaccine: opposed to views vs. in agreement with views; Only symptomatic cases spread COVID-19; Confidence about getting one or more vaccine shots as recommended by health professionals; Difficulty of hearing/reading things from others that discourage people from getting the vaccine |
| Caserotti, et al. 2021 [33] | **Predictors:** Intercept; Lockdown; Re-opening; COVID-19 Perc. Risk; Flu Perc. Risk; EVD Perc. Risk; Vaccine doubts 2nd quintile; Willingness to pay COVID-19 vaccine; Age; Gender; Deprivation index; Italian Area; Randomization order (Flu|COVID-19) |
| Chadwick et al. 2021 [34] | TV- dependent media diet |
| Chaudhary et al. 2021 [35] | lack of knowledge and understanding, and perception of the risk, safety, and comfort in administration= low rate of vaccine acceptance |
| Chen, et al. 2021 [36] | **negatively associated:** A mediating effect of self-efficacy, influenced by perceived barriers; perceived benefits; agreement with recommendations from authorities; agreement with recommendations from friends or family/**higher vaccine hesitancy:** female; nonmedical personnel; poor self-rated health; monthly salary over ¥10,000; a high level of perceived barriers; perceived benefits./**risk factors for vaccine hesitancy:** female gender; monthly salary over ¥10,000; poor self-rated health; high perceived susceptibility to COVID-19; high perceived barriers to vaccination/**protective factors against vaccine hesitancy:** occupation as medical personnel; high perceived benefits of vaccination; high self-efficacy for vaccination; agreement with recommendations from authorities; agreement with recommendations from friends or family/**Health Belief Model Factors (more):** high perceived susceptibility to COVID-19; high perceived barriers to vaccination/**Health Belief Model Factors (less):** high perceived benefits of vaccination; high self-efficacy for vaccination; agreed with recommendations from authorities; agreed with recommendations from friends or family; agreed with the vaccination of authorities; agreed with the vaccination of friends or family |
| Corcoran, et al. 2021 [37] | **negatively associated with COVID-19 vaccine confidence:** Christian nationalism. **positively and significantly associated with COVID-19 vaccine confidence:** income, education, age, political conservatism, political party, and religious service attendance./those who are never married have significantly higher levels of COVID-19 vaccine confidence./ Black, non-Hispanic respondents have significantly lower levels of COVID-19 vaccine confidence compared to White non-Hispanic respondents./In terms of religious tradition, Catholics, agnostics, and atheists have significantly higher levels of COVID-19 vaccine confidence compared to evangelical Protestants. |
| Cordina, et al 2021 [38] | **Predictors of willingness to take COVID-19 Vaccine:** gender; Knows about COVID-19; News and Information seen about COVID-19; I engage in preventative behavior; Believes that COVID-19 vaccines will help protect the health of the people who take it; Opinion of family and friends important in decision to take COVID-19 vaccine; Value the advice of healthcare professionals re effectiveness of COVID-19 vaccine; Is a health worker; Has a chronic condition; age; education level; Had flu jab last year; Unsure if give COVID-19 vaccine to children; Willing to give COVID-19 vaccine to children; Unsure if encourage COVID-19 vaccine to elderly parents; Willing to encourage COVID-19 vaccine to elderly parents**/ Reasons for refusing:** i do not think that it will give the necessary immunity; i have had a bad experience with vaccines; COVID-19 is just like any other flu that will pass; i am afraid of injections; i believe in natural and traditional remedies; i am against vaccination in general; i think COVID-19 vaccine may not be safe; Too short a time for development and testing (10 comments); Long term repercussions (2 comments); Do not trust system; Political game; I want to build my own immunity; Harmful substance in vaccine (2 comments); Vaccine not reliable; Have a condition/cancer (2 comments); Do not believe COVID is a threat; Vaccine will not help; Vaccine is a money-making venture; Not safe with lower efficacy; I am not a guinea pig/**Reasons for being unsure:** i have had a bad experience with vaccines; i am afraid of injections; i am not sure it will give the necessary immunity; i would want more information about the vaccine; i would want to see whether the number of infected; i am afraid that it will have unknown side effects; Planned pregnancy/fear of being pregnant (3 comments); Prefer vaccine that has been tested; Allergic to latex; Afraid of long-term effects (2 comments); Not convinced of number of infected reported; Never took vaccine before; Not convinced of the safety standards of vaccine; How long will immunity last?; Length of testing too short; Something that nobody knows about; Depends where it has been developed; Would like to know how effective it is |
| Cotfas, et al. 2021 [39] | **reasons:** Mistrust; Freedom; Side effects; Hiding relevant information; Unsafety; Inefﬁciency; existence of alternatives; Scam; Moral and religious issues |
| Danabal, et al. 2021 [40] | **highly mistrusting of the vaccines:** Younger individuals, women, rural residents, belonging to low income laborer class/**attitudes towards COVID 19 vaccines:** Mistrust in health system and COVID 19 vaccine; Trust in effectiveness of COVID 19 vaccine; Concerns regarding adverse effects of COVID 19 vaccines; Preference for Natural Immunity compared to Vaccines |
| de Sousa et al 2021 [41] | **higher prevalence of VH:** women; older adults; people with a high level of education; participants reporting perceived high stress; participants that have used early treatments in the presence of symptoms; people afraid of future repercussions of the disease; participants that have tested positive for COVID-19; and people in close contact with someone who had COVID 19 or died from the disease. /**reasons:** who reported agreeing with the statements “COVID-19 vaccines alter DNA”, “the vaccine can cause other diseases, such as autism or autoimmune diseases” or “the COVID-19 vaccine contains chips implanted to control people”/**direct effect for VH:** vaccine-related conspiracy beliefs; perceived stress; COVID-19 Misinformation; and individual responses to COVID-19 |
| Dinga, et al.2021 [42] | **determinants:** Communication and Media Environment, Perception of pharmaceutical industry, Reliability and/or source of vaccine and cost. |
| Doherty, et al. 2021 [43] | **Reasons:** disbelief vaccine works; safety concerns; mistrust government; mistrust medical system; want others to get vaccine first/ Factors associated: Race; Gender; Calendar time; safety concerns; government distrust |
| Dror, et al. 2020 [44] | **Positive predictors include** sex (male), and self-perception of disease risk. **/several positive predictors for vaccination:** status as a physician, employment in healthcare settings caring specifically for SARS-CoV-2 positive patients, unemployment during the quarantine period, and male sex; negative predictors include occupation as a nurse and parenthood/**Concerns of COVID-19 vaccines:** quality control; side effect; associated COVID-19 illness; wait until tested by others; wait for next year; pregnancy; doubted efficiency; COVID-19 symptoms are mostly mild; physiological immunity is better. |
| Du, et al. 2021 [45] | Region; Age group (years); Education; score on knowledge of COVID-19/**associated with:** lower risk perceived susceptibility; high risk perceived barriers; lower risk perceived beneﬁt |
| Duong, et al.2021 [46] | **Associated Factors:** gender; School type; WHO-5 Wellbeing Index, a 10-score increment; Coronavirus-related HL |
| Edwards, et al. 2021 [47] | **hesitant or resistant:** Females; those living in disadvantaged areas, those who reported that risks of COVID-19 was overstated, those who had more populist views and higher levels of religiosity |
| Ehde, et al. 2021 [48] | associated with having a lower level of education, being non-White, not having a recent flu vaccination, holding a lower perception of one’s risk of getting COVID-19, and having lower trust in the Centers for Disease Control and Prevention./ **reasons for and factors:** Efficacy; Vaccine approval process; Being first to receive new vaccine; Health conditions/medical history; Religious beliefs; Want others more at risk to receive vaccine first; Cost of/access to vaccine; Dislike of needles; Want additional information first; COVID is not a big risk; More worried about vaccine than COVID |
| El-Sokkary, et al. 2021 [49] | **Predictors of the intention:** Income; Years of experiences |
| Fedele, et al. 2021 [50] | **Reasons:** Fear of side effects; I don’t think it’s safe; I don’t think it’s useful; I am against vaccinations in general./ **associated with:** age; gender; Education; Job; Family cohabitants; Impact of COVID-19 on your everyday life; Impact of COVID-19 on your job; How important is receiving vaccine advice from the Ministry of Health |
| Fernandes, et al.2021 [51] | **Concerns:** record time; side effects; risk group concerns; in effective; microchip/**determinants of vaccination intention:** COVID-19 vaccine general beliefs and attitudes |
| Freeman, et al. 2020 [52] | **associated with:** age, gender, income, and ethnicity, lower adherence to social distancing guidelines/**Explanatory factor:** Importance of a COVID-19 vaccine; Beliefs that may get COVID-19 and the vaccine will work; Speed of vaccine development; Vaccine side effects; General knowledge about vaccines; Knowledge about childhood vaccines; Interpersonal disrespect by doctors; Respect from doctors; Negative views of vaccine developers; Positive attitude to doctors; Negative attitude to doctors; Positive attitude to medicine; Negative attitude to medicine; Social ladder relative to other people in the UK; Social ladder relative to other people in their community; Negative beliefs about the self; Positive beliefs about the self; Positive GP experiences; Positive NHS experiences; Negative NHS experiences; Coronavirus conspiracy beliefs (general); Vaccine conspiracy beliefs; Others disrespectful; Others react negatively; Anger; Need for chaos; Libertarian views; Populist views; Religious influence on health behavior; Illness as punishment for sin |
| Freeman, et al. 2021 [53] | **predictors:** fear of injection; Age; Ethnicity (Asian, Black, White other, Other background); income; Indirect effects and direct effects |
| Freeman, et al. 2021 [54] | factors of vaccine acceptance: awareness of collective benefit, provision of information on personal benefit |
| Fridman, et al. 2021 [55] | political affiliation |
| Gao, et al. 2021 [56] | **demographic characteristics:** family address, education level, training related to COVID-19 vaccines, and history of influenza vaccination./ **main factors associated:** knowledge about COVID-19 vaccine, training related to COVID-19 vaccines, family address, and education level/common reasons: worrying about the side effects of vaccines; uncertainty about the safety of vaccines; and underestimating the risk of exposure to COVID-19 |
| Gaur, et al. 2021 [57] | **Reasons:** Not-yet-decided; No need of vaccination; Fear related to vaccine side effects; Fear related to disease worsening; Distrust on vaccine; May not be effective/**Predictors:** age; education level |
| Geana, et al. 2021 [58] | **reasons:** not trusting the vaccine because of its pace of development or because they were concerned about long-term adverse effects; Opinions shared by family and friends |
| Gehlbach, et al. 2021 [59] | misinformation, lack of trust in institutions, and insecurity around employment and residency |
| Gerretsen, et al. 2021 [60] | **Sociodemographic factors associated:** younger age, women, race (i.e., Black participants), employment status (i.e., employed compared to retirees), and right-wing political status./**main determinants of vaccine hesitancy:** Lower population density; and lower household income./ **Complacency factors associated with:** lower perceived seriousness of COVID-19 and less health risk factors for COVID-19./**Confidence factors associated with:** mistrust in vaccine benefit, concerns about commercial profiteering, and preference for natural immunity./ Other psychological factors associated with: risk propensity, general mistrust in others, and negative impact of COVID-19 on mental health |
| Gerussi, et al. 2021 [61] | **Reasons:** I am concerned about the safety and/or the side effects; I am concerned because I don't think the vaccine will be effective; I don’t think I will need the vaccine due to previous infection, health status or age; I am against vaccines in general; I can’t take any vaccine because of previous vaccine reactions./**prone to undertake the SARS-CoV-2 vaccination:** people > 65 years, those who previously received influenza immunization and those working in contact with the public, Gender (women) |
| Griffith, et al. 2021 [62] | **concerning vaccine hesitancy:** concerns over safety, suspicion about political or economic forces driving the COVID-19 pandemic or vaccine development, a lack of knowledge about the vaccine, antivaccine or confusing messages from authority figures, and a lack of legal liability from vaccine companies |
| Harrison, et al. 2021 [63] | **reasons:** general concerns and personal concerns about vaccine safety and effectiveness, lack of trust in the vaccine effort, misinformation about the vaccine, and increasing vaccine uptake |
| He, et al. 2021 [64] | **Key contributing demographic factors:** income and race |
| Holeva, et al. 2021 [65] | Gender; Education; Age; Residence area; Profession; COVID-19 related beliefs |
| Hossain, et al. 2021 [66] | **predictors:** theory of planned behavior (Attitude toward COVID-19 vaccine, Subjective norm, Perceived behavioral control, Anticipated regret); the 5C psychological antecedents of vaccination (COVID- 19 vaccine conﬁdence, Complacency, more calculative about the pros and cons of getting vaccinated or needed more information about the vaccine, collective responsibility to vaccinate against COVID-19); the health belief model (perceived susceptibility, perceived severity, perceived beneﬁts, and perceived barriers, Social media (e.g., Facebook) or online news portals/blogs as a source of knowledge about the COVID-19 vaccine); |
| Hou, et al. 2021 [67] | Vaccine confidence: (Perceived importance of vaccines, Perceived effectiveness of vaccines, Perceived safety of vaccines, Trust in governments, Trust in experts, Information around vaccines); Complacency: (Perceived risk of getting COVID-19); Vaccine convenience (Vaccine types) |
| Hwang, et al. 2021 [68] | **reasons:** Confidence (I worried about side effects after vaccination. I think the vaccine for COVID-19 is not safe.); Complacency (If I were infected with COVID-19, it would be fine with getting treatment. Disease and life or deaths depend on fate. I will not be infected with COVID-19. I believe in natural healing and folk remedies.); others (I am afraid of getting an injection. I am against vaccination itself. I cannot get a vaccine for religious reasons.)/**Socio-demographic characteristics:** Family income per month; Job status; Health status; Religion/**Psychological and experiential characteristics:** Fears of COVID-19 infection; Being aware of the preventive guideline of COVID-19; Trust on preventive measures of the government; COVID-19 infection of oneself or acquaintances; Needs for medical care besides COVID-19; Changes of job status; Decrease of monthly family income; Changes in health status |
| İkiışık, et al. 2021 [69] | **statements about COVID-19 vaccine by vaccine intention:** Vaccination will be effective in preventing and controlling the disease; If the vaccine is easy to apply, I will have it; If vaccines are not paid, I will have it; If the vaccine does not have side effects, I will have it; I still get vaccinated even if I have had the disease; If there is a national COVID-19 vaccine, I will have it; If there is a vaccine from abroad, I will have it; I would like all my family members to be vaccinated; I just want my children to be vaccinated; I don't think COVID-19 disease is risky for my health; I think I will be protected from the disease in natural and traditional ways /**Factors affecting vaccine acceptance:** perception of risk; age |
| Jain, et al. 2021 [70] | **reasons:** concerned about safety of vaccine; concerned about efficacy of vaccine; not needed for young individuals like me; not needed as many people are now immune to the virus; not needed as COVID-19 pandemic is getting over now; not needed as I already had COVID-19/ **Determinants:** Studying in government medical college; Studying in clinical year; Male gender; Lack of awareness regarding eligibility of medical students for COVID-19 vaccination; Presence of risk perception regarding COVID-19; Prior vaccination experience present; Concern regarding adverse effect of vaccine; Concern regarding efficacy of vaccine; Lack of trust in govt. or public health authorities |
| Jennings, et al.2021 [71] | **Positive Factors for Vaccination Willingness:** age and trust in health organizations; who consume a large amount of information from traditional media; followed by positive evaluations of government handling of the COVID-19 crisis; trust in experts and government; social trust; Perceived personal threat of COVID-19; support for the governing Conservative Party; trust in information from the media; those with a graduate degree or above; who consume a large amount of information online/**Negative Factors for Vaccination Willingness:** conspiracy beliefs; distrust of vaccines, belief in COVID-19 misinformation, and ”lockdown skepticism”; General mistrust and distrust in government; Users of Instagram, YouTube, Snapchat, and TikTok; /**vaccine hesitancy is driven by:** a misunderstanding of herd immunity as providing protection, fear of rapid vaccine development and side effects, and beliefs that the virus is man-made and used for population control./**Reasons for decision to have the vaccine:** to protect their families and/or as their civic duty to protect society |
| Jin, et al.2021 [72] | **Vaccine Willingness:** the perceived threat of COVID-19/**Vaccine Hesitancy:** conspiracy theories |
| Joshi, et al. 2021 [73] | **contributor:** The lack of dependents at home/**reasons:** insufficient information regarding the vaccine; fear of unknown adverse effects; doubt in vaccine effectiveness; distrust in vaccine company; fear of vaccine's effect on current pregnancy |
| Khaled, et al.2021 [74] | **associated with hesitancy and resistance:** Arab ethnicity, migrant status/type, and vaccine side-effects concerns/**Willingness to Get the Vaccine:** Migrant Status/Type; Education Level; Gender; Age Group (Years); Employment Status; Ethnicity; Living Arrangement; Depression or Anxiety; COVID-19 Infection Concerns; Effective COVID-19 Containment in Qatar; COVID-19 Vaccine Side-Effects Are of Concern; COVID-19 Vaccine Should Be Mandatory; Endorsement Source for Vaccine |
| Khan, et al. 2021 [75] | women; younger./**associated with vaccine hesitancy among younger women:** age, age squared, having children, and subjective health status./**associated with vaccine hesitancy among older women:** age, age squared, and log of household income./**associated with vaccine hesitancy among the youngest subsamples:** having a spouse, university degree, subjective health status, and anxiety about the future./**associated with vaccine hesitancy among the 35–49 age group:** age, age squared, having children, and subjective health status./ **associated with vaccine hesitancy among the 50–64 age group:** subjective health status./**associated with vaccine hesitancy among younger men:** age, age squared, log of household income, log of household assets, ﬁnancial literacy, subjective health status, anxiety about the future, and myopic view about the future./**associated with vaccine hesitancy among older men:** having children, log of household income, and log of household assets./**younger men’s vaccine hesitancy:** age structure, ﬁnancial literacy, subjective health status, anxiety about the future, and myopic view about the future. |
| King, et al. 2021 [76] | occupation category/**Reasons:** Side effects; Don't trust COVID-19 vaccine; Wait to see if safe then maybe later; Don't trust government; Don’t believe I need; Allergic reaction; Don't know if it will work; Other people need more; Don't like vaccines; Safety concern because of health condition; Doctor not recommended; Currently/planning to be pregnant/ breastfeeding; Against religion; Cost |
| Knight, et al. 2021 [77] | **Common reasons for vaccine hesitancy (WHO 3C category):** Confidence(Concerns about unknown long-term effects, Concerns about side effects, Concerns there is an insufficient testing/evidence base, Concerns the development of the vaccine has been rushed, Concerns about the safety of the vaccine (but not explicitly side effects), Unsure about vaccine effectiveness, Concerns around vaccine interactions/ effectiveness with existing conditions, Lack of trust in the manufacturer/government/scientists etc.,; Complacency (Believe they are not at high risk of COVID-19, Believe they are in good health / Their body can fight off the virus, Have already had COVID-19; Convenience (Other people need it more, Lack of knowledge about the vaccine, Don’t like injections/vaccine experience, Inconvenience, Freedom of choice./**reasons for vaccine acceptance:** Self-protection; Protect specific others (e.g., family, friends, colleagues etc.);Protect the population/non-specific others and control the virus; Confidence in SARS-Cov-2 vaccine; Hope to end the pandemic/ wish for normal life; Civil duty/Requirement; Non-specific pro-vaccine/pro-science statement./**most common reasons for vaccine hesitancy:** “I don’t know if the vaccines have been tested on people like me: By age, ethnicity, and comorbid health condition”; “I don’t think we know enough about the side-effects of the vaccines”; “I think the whole process has been rushed”; “I don’t know if they will work”; “I don’t think I am at risk of getting COVID-19”; “I think my body can fight the virus on its own”; “I just don’t know enough about it: Safety and effectiveness concerns”; “Other people need it more than me”; “I’m worried I would have to wait 12 weeks before I get my second dose”. |
| Kose, et al. 2021 [78] | Factors associated with the willingness of to get the COVID-19 vaccine: Profession; previous flu vaccination status; age groups; gender; the desire to get COVID-19 vaccine./reasons: they think the vaccine may have side effects, they do not trust the vaccine because it is a new one, they do not believe the vaccine will work, they trust their own immune system, they do not need to be vaccinated because they are protected from the disease, and they are not afraid of getting sick. |
| Kumar, et al. 2021 [79] | **predictors:** female gender, concerns about vaccine safety, safety after the vaccination, and doubts about the vaccine’s protection/**associated with:** age |
| Kwok, et al. 2021 [80] | factors: (i) individuals may lack confidence in and be fearful towards vaccines, especially with the misunderstanding that vaccines pose a risk of infection; (ii) individuals do not perceive a need for a vaccine (e.g. due to under estimation of disease severity) or do not value the vaccine; and (iii) individuals or community may have difficulties accessing the vaccine (WHO)./vaccination intention was associated with younger age, more confidence, less complacency and more collective responsibility. COVID-19-related demands were associated with greater work stress, and hence stronger COVID-19 vaccination intention./Univariate factors associated with stronger intention to take COVID-19 vaccine were stronger vaccine confidence, calculation, collective responsibility, and work stress; and weaker complacency and constraints. |
| Lamot, et al. 2020 [81] | **predictors:** Age; Education; Employment status; Health; Political orientation |
| Li, et al. 2021 [82] | **predictors:**  medical occupation; little or no trust in COVID-19 vaccine information from physicians/other providers; being age 30 or younger. |
| Liu, et al. 2021 [83] | **Associated factors:** gender; age; risk of infection COVID-19; eHealth Literacy |
| Liu, and Li, .2021 [84] | **factors:** trust in modern science, mainstream medicine, health authorities, large corporations, and government; concerns about side-risks or other perceived risks, and the preference for “natural risks” over “manmade risks”; access to health information through health professionals and social networks ; misinformation and misperceptions ; “local vaccination cultures” that characterize shared local beliefs, views, and vaccination settings; past experiences with vaccination; philosophical, moral, or religious convictions/State partisanship had a strong correlation with state percentage of people with vaccine hesitancy/significant racial differences in hesitancy/gender differences |
| Liu, et al.2021 [85] | **factors:** Sex; Age interval in years; Highest educational level; Occupation and working area; Annual salary level; Acceptance of vaccination; Ever been infected with COVID-19; Friend family or community ever infected; Marital state; Source of information of COVID-19 vaccines./**The relative importance of COVID-19 vaccine attributes:** The cost of vaccination; Vaccine varieties; Adverse effect; Efficacy; The duration of vaccines. |
| Lockyer, et al. 2021 [86] | **factors:** safety concerns, Negative stories and misinformation focusing on the vaccine and personal knowledge |
| Lucia, et al. 2021 [87] | **factors:** concerns about serious vaccine side effects and lack of trust in the information received from public health experts. need for transparency and concerns about the speed of vaccine development potentially impacting vaccine safety |
| Mahdi, 2021 [88] | **vaccine refusal:** gender; Age; 1–3 stage pre-clinical; 4–6 stage clinical; Province Baghdad |
| Mangla, et al. 2021 [89] | education level; age; country of residence |
| Maraqa, et al. 2021 [90] | **Factors predicting:** sex; age; income level; profession; health care setting type; influenza vaccine uptake; patients contact per day; perceived COVID-19 knowledge; infected with COVID-19; COVID-19 perceived susceptibility; COVID-19 perceived severity; Vaccine perceived benefits; Vaccine perceived barriers. **variety of triggers that may influence their intention to receive the vaccine:** I'll be more optimistic about the COVID-19 vaccine if I know more about it; I will be more confident if experts or people I trust would recommend the vaccine; the type of vaccine that is available would affect my decision; COVID-19 vaccines are being misrepresented in the media |
| McElfish, et al. 2021 [91] | **Sociodemographic Determinants:** age, sex, race, and education |
| Mejri, et al. 2021 [92] | **predictive factors: REFUSE:** patients who think that the vaccine may interfere with treatment efficacy; may impact cancer outcome; **REJECT:** Patients who disagree that the vaccine is a major weapon against the pandemic; or that it could reduce the virus transmission; **AGAINST:** Confidence level in the authorities; indeed patients who are not registered; not informed about the Tunisian national vaccination platform EVAX ; |
| Mohan, et al. 2021 [93] | **factor determining:** Qatari nationals; vaccine specific safety concerns; ethnicity**/Attitudes, knowledge and beliefs around COVID-19 vaccination**: Covid is not a real disease; Covid-19 vaccine not safe; I feel safe after vaccination; I rely on vaccines to stop serious infectious diseases; I feel protected after vaccination; Although vaccines appear safe there may be problems; Vaccines may cause problems in children; I worry about vaccine effects in the future; Vaccines make a lot of money for pharmaceutical; Authorities promote vaccines for financial gain but not for; Vaccines are a big conn; Natural immunity lasts longer; Natural immunity is the safest protection; Being exposed to diseases is safer then vaccination |
| Mollalo, and Tatar, 2021 [94] | **COVID-19 vaccination rate associated with:** Per capita income and Minority; Age 17 and younger; Mobile homes; Uninsured people |
| Momplaisir, et al.2021 [95] | **factors:** Race/ethnicity; Sex; Age; Education level; Area of residence; Employment length.**/Reasons for COVID-19 Vaccine Hesitancy:** Concern about side effects; Vaccine is too new; Don’t know enough about the vaccine; It may not work; Concern about getting infected with COVID-19 from the vaccine; Not concerned about getting seriously ill from COVID-19; I do not like vaccines; COVID-19 outbreak is not as serious as some people say it is; I do not like needles; I won’t have time to get vaccinated./**Reasons for COVID-19 Vaccine Acceptance:** Want to protect my family; Want to protect myself; Want to protect my community; Life won’t get back to normal until most people are vaccinated; Want to prevent getting seriously ill from COVID-19; Allow me to feel safe around other people; I want to travel again; My employer recommends the vaccine; I have a chronic condition, so it is important I am vaccinated; Doctor recommends the vaccine. |
| Monami, et al. 2021 [96] | **correlated with:** Age; Live with subject/s aged ≥ 65 years; prior SARS-CoV-2 infection; diabetes; Adverse Events at previous vaccinations and refusal of 2020 flu vaccine/**reasons:** concerns about vaccine Adverse Events |
| Moore, et al. 2021 [97] | **factors associated with vaccine hesitancy:** assigning importance to the vaccine´ s efficacy; fear of adverse reactions; assigning importance to the vaccine´ s country of origin.**/ risk factors:** Gender; Age bracket; Schooling; Family income; Children; Residence in state capital. |
| Moore, et al. 2021 [98] | **Likelihood of Vaccine Resistance:** those under 30 and those experiencing housing insecurity attributed to the COVID-19 pandemic; gender; Current health insurance; Current Tobacco Smoker; Median number of comorbidities/**determinant of vaccine hesitancy:** age; gender; Employed full-time; Current Tobacco Smoker; Median number of comorbidities |
| Muhajarine, Net al. 2021 [99] | **factors:** lower education level, financial instability, Indigenous status, and not being concerned about spreading the coronavirus. /**unsure about getting vaccinated:** gender; Place of residence |
| Muric, et al. 2021 [100] | **predictor of vaccine hesitancy:** political orientation /**reasons:** misinformation originating in Websites with questionable credibility |
| Murphy, et al.2021 [101] | **demographic factors:** sex, age, and income level**/psychological indicators:** Altruism(Identify with others; Care about others; Help others); Personality(Extraversion); LOC(Internal); CRT(Tests 1-3); Beliefs (Religious; Conspiracy); Trust (State; Scientists; Health care profs); Socio-political views (Authoritarianism; Social dominance; Migrant views 1; Migrant views 2)/**Sociodemographic, political, and health indicators:** Ethnicity; Income; Underlying health condition./**vaccine hesitant:** female; between 35 and 44 years; less likely to have received treatment for a mental health problem; |
| Musa, et al.2021 [102] | **Inﬂuencing Factors of Vaccine Hesitancy Rates:** age groups; nationalities; and recovery from COVID-19; Chronic Disease |
| Navarre, et al. 2021 [103] | **predictive factors:** gender; age; profession; sector/Distrust of health authorities and pharmaceutical lobbying were the main **obstacles to vaccination**./**reasons:** I think I will not develop severe Covid-19; I already had Covid-19; mistrust of pharmaceutical lobbies; mistrust of health authorities |
| Nazlı, et al. 2021 [104] | positive correlation with belief in conspiracy theories; low fear of COVID-19 |
| Nguyen, 2021 [105] | Race/Ethnicity (Black; Hispanic or Latino)./**reasons:** Vaccine is too new; concerns about side effects; newness of the vaccine; lack of vaccine knowledge; It may not work; Concern about getting infected with COVID-19 from the vaccine; Not concerned about getting seriously ill from COVID-19; I do not like vaccines; COVID-19 outbreak is not as serious as some people say it is; I do not like needles; I won’t have time to get vaccinated |
| Okoro, et al. 2021 [106] | **Vaccine hesitancy:** Vaccine Compliance: **(**High-risk perception, Influence of peer-pressure, Sense of communal health and safety, Anticipation of policy mandate [e.g., for travel], Fear fatigue [tired of living in fear of infection])**)**; Vaccine complacency: **(**Low-risk perception, Continued effectiveness of preventive behaviors, Preference for alternative preventive treatments**);** Vaccine confidence: **(**Fears/concerns, Vaccine literacy, Perceived efficacy and safety per timeframe of vaccine development and Demonstrated efficacy in others**);** Vaccine “resistance” (actively against vaccine): **(**Conspiracy theories, Confecting beliefs (e.g., religious), Distrust of Government (perception of political agenda), Perceived trustworthiness of healthcare**)** |
| Okubo, et al.2021 [107] | **factors associated:** sex; age; income; Marital status; living alone; occupation; educational level; alcohol use; presence of comorbidities (diabetes mellitus, cardiovascular disease, cancer, chronic pain, psychiatric disorder); personal history of COVID-19 infection; fear of COVID-19-induced death; distrust toward the government; the thought of embarrassment of getting infected with COVID-19; and presence of severe psychological distress/**Reasons for getting vaccinated:** It was recommended by a family member or friend It was recommended by SNS or the media; I’m worried about getting infected with COVID-19 I think I have a high risk of becoming seriously ill; I am a medical worker; I don’t want to infect my family or other people around me.; I think it is necessary for society to be vaccinated; I can get it for free/**Reasons for not getting vaccinated:** I don’t have time to go get vaccinated I’m worried about adverse reactions; I don’t think it is very effective I don’t think I will get infected; I think I have a low risk of getting seriously ill; I was previously infected with COVID-19; I have already received the COVID-19 vaccine It was recommended by a family member or friend; It was recommended by social network services or the media. |
| Oliveira, et al. 2021 [108] | **Factors associated:** Cities stratum; Age group; Gender; Religion; Frequency of symptoms possibly related to covid-19 |
| Palamenghi, et al.2020 [109] | **willingness to vaccine:** age; trust in scientific research; general attitude towards vaccines’ efficacy |
| Park, et al. 2021 [110] | **vaccine hesitancy was higher:** women, younger participants, those who perceived lower levels of risk from COVID-19, those who doubted safety of a COVID-19 vaccine, and those who perceived the government’s COVID-19-related countermeasures as ineffective or inappropriate./**factors:** age, political belief, affective risk perception of COVID-19, cognitive risk perception of COVID-19, perceived safety of a COVID-19 vaccine, and perceived performance of the government’s COVID-19-related countermeasures |
| Prickett et al.2021 [111] | **Reasons for vaccine hesitancy:** Worried about unknown future effects of the vaccine; Worried about side effects; Chances of me becoming seriously unwell are low; I don't trust vaccines; Vaccines are limited and others need it more than me; Impact of COVID-19 is greatly exaggerated; Have a condition which would make it unsafe for me; Don't think it's effective at stopping me catching COVID-19; Herd immunity will protect me; I don't have time; Advised by a health/medical professional not to get the vaccine/**Reasons for likelihood of COVID-19 vaccine uptake:** To stop me catching COVID-19 or getting very ill from it; Protect other people from catching COVID-19; Help my community to get back to normal; Vaccine won’t work unless most people in NZ get it; Help my social and family life to get back to normal; I take the vaccines offered or recommended to me; I am a key worker working with high risk groups./**predictors:** Age; Gender; Educational attainment |
| Puteikis, K. and Mameniškienė, R. 2021 [112] | **Willingness to be vaccinated was associated with:** receiving an influenza shot; the beliefs that vaccines are generally safe; they are the only convenient way to gain immunity. **factors associated with the willingness to be vaccinated:** Prior vaccination history, positive outlook on the safety profile of vaccines and an understanding of how they function/**hesitant:** they thought it could cause the infection; related to erroneous beliefs about their safety and mechanism of action |
| Qunaibi et al. 2021 [113] | **reasons:** concerns about side effects and distrust in health care policies, vaccine expedited production, published studies and vaccine producing companies./**factors:** gender; age; Chronic Diseases; Academic Education; Vaccine type unknown./**Barriers to COVID-19 vaccine acceptance:** Afraid of unknown side effects; Do not think I will get COVID19; I have allergies to foods/drugs; I am not eligible (pregnant or <16 old); I have a chronic disease; May get COVID19 after Vaccine; I do not like needles; Most people already had COVID; Vaccines were not tested in Arabs; Most vaccinated people had SE; Vaccines contain Aluminum; Infection rate decreasing; I had/have COVID; Do not believe in vaccines in general; Vaccine immunity is short; Vaccine may cause death; Afraid of SE mentioned in studies; Vaccine can cause COVID19; Coronavirus/vaccine are conspiracy; No published studies on vaccine; Vaccines irreversibly alter DNA; Most infected people recover; No value for new strains; Pandemic is exaggerated to benefit pharma; Insufficient numbers on studies; Do not trust company/studies; Do not trust the healthcare policies; Vaccine production was rushed; Not enough time to test vaccine safety |
| Ramonfaur, et al. 2021 [114] | **higher hesitancy:** having a professional degree, belonging to the middle- or high-income category, being catholic, having rejected a vaccine before due to fear, having had a serious adverse effect attributed to a previous vaccine, and taking supplements with the purpose of preventing COVID-19 infection./**higher vaccine acceptance:** being male, having any comorbidity, living with someone older than 60 years of age, and having been vaccinated against influenza in the past three years./**rejecting:** Having private insurance |
| Reno, et al. 2021 [115] | **associated with:** information sources; educational level; age; income./ **predictors:** Past vaccination refusal; perceived risk of infection; presence of comorbidities |
| Reno, et al.2021 [116] | **Predictors of vaccine hesitancy:** Employment status; Family income; Income reduction due to pandemic; age; gender; education; comorbidities; past vaccination refusal; and perceived risk of infection/**reasons for past vaccine refusal:** I did not think it was needed; I did not have enough information on the vaccine; I did not think the vaccine was effective; I did not think the vaccine was safe; I was worried about side effects; I had a bad experience with a previous vaccination; Logistical issues |
| Riad, et al. 2021 [117] | **Predictors of vaccine acceptance:** Trust in the pharmaceutical industry; trust in healthcare providers; and perceived knowledge/**determinants of vaccine acceptance:** University; Field of Study; Medical and Healthcare Faculties./**determinants of vaccine hesitancy:** gender; age; nationality; Media and Social Media; healthcare students; insufficient knowledge about vaccine safety; misconception about immunity; decision upon the safety surveillance |
| Riad, et al. 2021 [118] | **higher levels of vaccine hesitancy:** low- and lower-middle-income (LLMI) economies**/Drivers of COVID-19 Vaccine Acceptance:** the dependence on media and social media to inform vaccine-related decision; the reliance on public figures and opinion leaders; confidence in government; the confidence in pharmaceutical companies; cultural and religious values; prevented by natural immunity than by vaccines; insufficient knowledge about COVID-19 vaccine safety; who did not think that the vaccine’s benefits outweigh its reported side effects; the students who were not inclined to take newly introduced vaccines; and the students who were not confident in finding the vaccine in their local health center when needed./**barriers to vaccination:** media and social media, public figures, insufficient knowledge about vaccines, and mistrust of governments and the pharmaceutical industry |
| Robertson, et al. 2021 [119] | **variables:** gender, age, ethnicity, Birth Country, Shielding, UK Country of residence, education level. **/reasons:** unknown future effects of the vaccine; side effects; vaccines are limited and other people need it more than me; trust vaccines; the chances of me becoming seriously unwell from the coronavirus are low; the chances of me catching the coronavirus are low; the impact of the coronavirus is being greatly exaggerated; i don't think it would be effective at stopping me catching the coronavirus; i don't think i would be offered the vaccine for free and i wouldn't pay for it; i have a condition which would make it unsafe for me; herd immunity will protect me even if i don't have the vaccine |
| Rodriguez 2021 [120] | **hesitation to vaccinate was higher:** those unwilling to receive the COVID-19 vaccine despite provider recommendations and among those anticipating being required to receive it for daily activities (e.g., work, travel)./Participants who believed that their health conditions would make it unsafe for them to receive the vaccine had greater hesitancy, as did participants who did not perceive that their health conditions made it important for them to receive the vaccine. participants who believed that they would not receive a required vaccine showed greater hesitancy scores. |
| Rozek, et al. 2021 [121] | Women; Older; highest level of economic wellbeing; country/territory./**predictors:** conﬁdence in the WHO and trust in health practitioners./**predictive of reduced vaccine hesitancy:** Measures of trust in scientists, local health departments, and national health ministries./Higher trust in religious leaders was predictive of increased vaccine hesitancy |
| Sadaqat, et al. 2021 [122] | **factor:** lack of confidence, risk factor concern, and misinformation |
| Saied, et al. 2021 [123] | **Significant:** Academic year/**Vaccine acceptance:** /**Vaccine refusal:** Adequacy of the available safety data for the new vaccine; COVID‐19 infection in close social network; Self‐perception of own health status/**barriers of COVID‐19 vaccination:** insufficient information regarding adverse effects of the vaccine; insufficient information regarding the vaccine itself; financial cost hindrance if the vaccine is not free; insufficient trust in the vaccination source |
| Sallam, et al. 2021 [124] | **factors affecting acceptance of COVID-19 vaccines:** Male sex, higher educational level and history of chronic disease, higher monthly income, related conspiracy beliefs, Country of residence |
| Savoia, et al. 2021 [125] | **determinants of vaccine hesitancy:** Gender; Education; Race; Risk perception; COVID-19 diagnosis; Experience of unfair treatment |
| Schernhammer, et al. 2021 [126] | **factors:** Gender; Region of residence; Political party preference; Optimism; age; education level; Area of residence; Resilience; Need for cognitive closure; Main source to inform oneself about measures/recommendations; Frequency of informing oneself; Perceived risk of infection between June–October 2020; Subjective health status; Overall adherence to measures; Change in quality of life. correlation between vaccine hesitancy, unwillingness to follow Corona measures and political apathy or opposition |
| Schwarzinger, et al.2021 [127] | **associated with:** female gender, age, lower educational level, poor compliance with recommended vaccinations in the past, and no report of specified chronic conditions (ie, no hypertension [ for vaccine hesitancy] or no chronic conditions other than hypertension [ for outright vaccine refusal]). Outright vaccine refusal was also associated with a lower perceived severity of COVID-19, whereas vaccine hesitancy was lower when herd immunity benefits were communicated and in working versus non working individuals, and those with experience of COVID-19 (had symptoms or knew someone with COVID-19). |
| Sharma, et al. 2021 [128] | **determinant:** age (younger Blacks being more hesitant), being from the Northeast region of the United States, being a Republican as political affiliation, and having a religion other than Christianity or atheist had statistically significant greater proportion of vaccine-hesitant Blacks. |
| Silva, et al.2021 [129] | related concerns were safety, effectiveness , and limited information. |
| Soares, et al.2021 [130] | **factors were associated with both refusal and delay to take the vaccine:** contextual factors: younger age and loss of income during the pandemic; individual and group factors: no intention of taking the flu vaccine this year; COVID-19 influences: low confidence in the health service response during the pandemic, worse perception of the adequacy of measures implemented by the government, and perception that the information provided by health authorities during the pandemic was inconsistent and contradictory; and COVID-19 vaccine-specific factors: low confidence in the COVID-19 vaccines being developed and answering the questionnaire before the release of information regarding the safety and efficacy of COVID-19 vaccines./**factors of intention of delaying intake of the COVID-19 vaccine (increase):** contextual factors: being female; individual and group factors: intention of taking the flu vaccine this year; and COVID-19 influences: unclear perceived risk of developing severe disease following COVID-19 infection./**factors of intention of delaying intake of the COVID-19 vaccine (decrease):** contextual factors: being a student./**factors of refusing intake of the COVID-19 vaccine (increase):** contextual factors: having lower levels of education; individual and group factors: having school-age children; and COVID-19 influences: low or nonexistent perceived risk of getting COVID-19 infection or developing severe disease following the infection./**factors of refusing intake of the COVID-19 vaccine (decrease):** contextual factors: being retired and individual and group factors: perception of worse health status and having comorbidities. |
| Sowa, et al.2021 [131] | **predictors of increased odds of vaccination:** age; gender; education; Place of residence; Parents education; Difﬁculty in paying bills in the last 12 months; Household debt; Fitness Index; Social Distance Index; Loneliness index; Concern about the side effects |
| Thaker, 2021 [132] | **Intension to get a COVID-19 vaccine:** Sex; Age; Education; Ethnicity; Annual personal income; Parental status; Smoking status; Trust in experts and general/**factors of vaccine hesitancy:** risk perceptions; lack of confidence in vaccines./**dimensions of vaccine hesitancy scale:** Vaccines are important for my health; Vaccines are effective; Being vaccinated is important for the health of others in my community; All vaccines offered by the government programme in my community are beneficial ; New vaccines carry more risks than older vaccines; The information I receive about vaccines from the vaccine program is reliable and trustworthy; Getting vaccines is a good way to protect myself from disease; Generally, I do what my doctor or health care provider recommends about vaccines; I am concerned about serious adverse effects of vaccines; I am uncomfortable getting a vaccine that was rushed into production; I feel uncomfortable getting vaccinated; Government over hypes the need for vaccines; Corporations manufacturing vaccines only care for profit; Vaccines cause diseases |
| Thanapluetiwong, et al. 2021 [133] | **factors (higher vaccine hesitancy):** vaccine manufacturers; low education; lack of confidence in the healthcare system’s ability to treat patients with COVID-19; being offered a vaccine from an unexpected manufacturer; a low number of new COVID-19 cases per day/**reasons:** fear of COVID-19 vaccine-related adverse effects; possible complications caused by an underlying disease; lack of confidence in COVID-19 vaccine efficacy or quality; the news regarding COVID-19 has been exaggeratedly terrible; observe potential adverse effects from others; waiting for a vaccine that is currently unavailable; chance of developing a severe infection are low; i was unable to detect my abnormal symptom as a result of vaccination; having a low risk of getting infection because i rarely go outside or interact with others; i could sufficiently protect myself from infection |
| Thelwall, et al. 2021 [134] | vaccines would contain microchips to monitor or control the population, they were a plot to depopulate the world, or that they were part of an unspecified secret Bill Gates plan./Focuses on whether vaccines may be a threat/risk to Black people./fears that vaccines had been rushed through the approval process and might therefore be unsafe because they had not been fully tested./ingredients of vaccines sounded worrying/was pointless to take the vaccine because its side-effects were like (mild) Covid-19 or that the tweeter was in a low risk category that did not need to be protected. |
| Tram, et al. 2021 [135] | **predictors:** Age, Female sex, Race, Hispanic ethnicity, Married, Income, education, and state political leaning, Survey period/ **reasons:** I don't think vaccines are beneficial; don't trust COVID-19 vaccines; don't trust the government; I don't believe COVID-19 is a serious illness; i don't believe I need it-Other; don't like vaccines; I plan to use masks or other precautions instead; I am not a member of a high-risk group; I already had COVID-19; Don't know if a vaccine will work; doctor has not recommended it; possible side effects; concerned about the cost; other people need it more right now; plan to wait and see if it is safe |
| Trent, et al. 2021 [136] | **reasons not to take the COVID-19 vaccine:** COVID-19 vaccine will not be safe; COVID-19 vaccine development is "rushed"; COVID-19 vaccine will not be effective; Low perceived risk of disease; Preference to take other measures or strengthen immune system; Distrust government or pharmaceutical industry; distrust of COVID-19 vaccine or vaccination in general/**Demographic and health predictors of intention to vaccinate against COVID-19:** Age; gender; Tertiary degree; Born overseas; Private health insurance; income; Current daily smoker; 1 or more chronic health condition; Influenza vaccine in previous 12 months. |
| Tsai, et al. 2021 [137] | **Hesitancy was increased with:** history of prior COVID-19; conservative political leaning; younger age; lower education level./**Positive predictors of vaccine acceptance:** routine influenza vaccination; trust in responsible vaccine development; residing in the USA; never smoker status |
| Turhan, et al.2021 [138] | health care system distrust and health literacy |
| Uhr, and Mateen, .2021 [139] | **WHO SAGE Vaccine Hesitancy Scale item:** I am concerned about serious side effects of vaccines; vaccines are important for my health; getting vaccinated is important for the health of others in my community; vaccines are effective; generally I do what my doctor or health care provider recommends about vaccines for me; getting vaccines is a good way to protect me from disease; new vaccines carry more risks than older vaccines; all vaccines offered by the government program in my community are beneficial; the information i receive about vaccines from the vaccine program is reliable and trustworthy; i do not need vaccines for diseases that are not common anymore. |
| Umakanthan, et al.2021 [140] | **reasons for vaccine hesitancy and resistance:** Post-vaccine adverse health effect; Existing co-morbidities; Lack of clarity in vaccine; Prefer frontline COVID-19 workers to receive complete vaccination; Lack of trust in vaccine protection; Had recent exposure to COVID-19 through close contacts; Spread of rumors through social media; Scare of vaccine administration; Prefer more concrete evidence on vaccine protection; Short-time period to decide; Want to wait for more effective vaccine/**determinants of vaccine hesitancy:** gender; age; education degree; Society variation; House-hold income/**determinants of inclined towards the vaccination:** higher income levels, lived in a society, maintained social distancing, had downloaded the COVID-19 update app, showed a positive attitude towards their government, and more confidence in their healthcare system |
| Uzochukwu et al. 2021 [141] | **Reasons:** Efficacy concern; Safety concern; disbelief over the existence of COVID-19; poor knowledge of COVID-19 vaccine; belief that other preventive measures are enough protection; belief that the body’s immunity is strong enough against the virus; the vaccine not needed if one is not infected with the virus; COVID-19 can be easily treated with medicines such as chloroquine; unpredictable consequences of messenger RNA (mRNA) COVID-19 vaccine; religious beliefs. **Factors influencing willingness to receive COVID-19 vaccine:** Age; Marital status; Church denomination; |
| Vallée, et al. 2021 [142] | **predictive of COVID-19 vaccination hesitancy:** General attitudes to vaccine: Have you ever refused vaccination for reasons other than an illness or an allergy recommended by your doctor?; COVID-19 – personal opinions: Do you have concerns about your health about COVID-19?; Personal views—COVID-19 and vaccines: – COVID-19 vaccination should be mandatory, Vaccination against COVID-19 is important to me as a patient with chronic disease, I am concerned about the serious side effects of a COVID-19 vaccine, I think I am immune to COVID-19 (because a serologic test has revealed the presence of antibodies) |
| Vergiev, S. and Niyazi, D. 2021 [143] | gender; Scientific area/ side effects |
| Walker, et al.2021 [144] | **barriers to COVID-19 vaccine acceptance:** safety; skepticism about efficacy; feeling rushed; and confusion about contradictory and changing COVID-19 information. |
| Wang, et al. 2021 [145] | **reasons**: worry about the safety of COVID-19 vaccine; low efficacy of the COVID-19 vaccine; it's safe in the country and no need to be vaccinated at this moment; the leaders and colleagues do not take the COVID-19 vaccine; I am healthy enough and no need to receive the COVID-19 vaccine; the relatives and friends do not support the COVID-19 vaccination./**predictors:** gender; age; education; Occupations; confidence |
| Wang, et al. 2021 [146] | **willing to accept the vaccine:** occupation; age; sex; previous inﬂuenza vaccine uptake; marriage; chronic condition/**Reasons for COVID-19 vaccine refusal or hesitancy:** doubt of effectiveness; vaccine is unnecessary; no time to uptake; worried about safety |
| Wang, et al.2021 [147] | **COVID-19 vaccine willingness was associated:** educational level/ **reason for unwillingness to be vaccinated:** concern about side effects/**predictors of vaccine hesitancy:** Age group; Relationship with child; Firstborn; Annual household income |
| Wang, et al. 2021 [148] | black race; having been tested for COVID in the past decreased./**Concerns of COVID Vaccine Among Respondents Who Expressed Vaccine Hesitancy:** I’m allergic to vaccines; I don't like needles; I'm not concerned about getting sick from COVID; I'm concerned about side effects; I don't think vaccines work well; I don't trust that the vaccine will be safe; I don't believe the covid pandemic is as bad; I don't want to pay for it; I don't know enough about how well the vaccine works |
| West, et al. 2021 [149] | **predictors:** Demographics Location; Married; age; married; education; immigration; health; Threat Perception & Risk Mitigation; Risk Exposures; Information & Communication; Follow News Everyday |
| Willis, et al. 2021 [150] | Age; sex; Race/ethnicity; Income; Education |
| Wong, et al.2021 [151] | **barriers to acquire vaccination:** Having heard of cases with serious adverse events or death after vaccination; Lack of confidence in the vaccine manufacturer and its country of origin; Waiting for a better vaccine” as barrier to you to get vaccinated; Confusing information about vaccines; Lack of confidence in governmental recommendations; Lack of confidence in the efficacy of vaccine; Inconvenient in getting to vaccination venue; Current health condition not suitable” as a barrier to you to get vaccinated |
| Xu, et al. 2021 [152] | **risk factors for vaccine hesitancy:** yearly household incomes; medical workers; general attitudes of Parental Attitudes toward Childhood Vaccines; behavior; safety and efﬁcacy.**/ reasons:** unpleasant vaccination experience; concerns over side effects of the vaccine; doubt about the effectiveness of vaccination; worried about unknown effects of the vaccine; relying only on protection from innate immunity; no risk of infection or no severe illness after infection; special physical conditions not suitable for vaccination; lack of access to vaccination related information; no time because of parents' busy work; vaccination conspiracy theories./**Protective factors:** female; children raised; rural residence |
| Xu, et al. 2021 [153] | **negatively correlated:** confrontation and avoidance coping styles/**predictors:** adoption of the coping styles of confrontation and avoidance; the residence of the patient |
| Yang, et al.2021 [154] | **Acceptance of COVID-19 vaccine:** Gender; Race /ethnicity; Educational attainment; Household income; Family size; Urbanization; If smoking; If stressed because of COVID-19 pandemic; Prior influenza vaccination |
| Yilmaz, et al.2021 [155] | **Reasons for Vaccine Hesitancy:** Fear/lack of confidence (Pregnancy, Breastfeeding, Desire to delay vaccination, Fear of side effects); Complacency (Being out of the risk group); Inconvenience in accessing vaccines (Time issues /inability to create time); |
| Zhang, et al. 2021 [156] | positivity: the reported information about the side effects of the COVID-19 vaccine or other vaccines in general; gender (male); regional epidemics arose |
| Zhuang, et al. 2021 [157] | **associated with lower willingness to be vaccinated:** patients with higher Comparisons of score of vaccine misconception; gender (female); concerns to underlying diseases in patients with lung cancer |
| Siegler, et al. 2021 [158] | willingness was higher: bachelor’s or graduate degree; Race/ethnicity |
| Bass, et al. 2021 [159] | associated with: race; income; Inattention to COVID news; satisfaction with health; healthcare access |
| Carcelen et al.2021 [160] | vaccination hesitancy was correlated with: beliefs around COVID-19 severity and risk, as well as vaccine safety and effectiveness |
| Ebrahimi, et al. 2021 [161] | predictors: Sex; Has children below 18 years; rural residents; Media preference; Perceived risk of vaccination, belief in the superiority of natural immunity, fear concerning signiﬁcant others being infected by the virus, and trust in health ofﬁcials’ dissemination of vaccine-related information |
| Khubchandani, et al.2021 [162] | Vaccine refusal: Area of Residence; Ethnicity; Education; Marital Status; Political Affiliation; COVID-19 infection among family/friends; COVID-19  hospitalization of family/friends; COVID-19 related death in family/friends; |
| Berry, et al.2021 [163] | concerns: Early concerns raised consistently (The vaccine was developed too quickly, Short term side effects, Infertility and safety in pregnancy, Long-term side effects, Wait and see how others react to the vaccine); Consistent concerns raised after early concerns were addressed (Belief that it causes COVID-19; Bell's palsy, Requirement for a booster shot, Ineffective in new COVID-19 variants, Previously tested positive for COVID-19); Concerns that were usually raised but later in discussion (Uncertainty as to whether getting vaccinated will change precautions, Microchip, Safety in persons with chronic disease); Infrequent concerns (Mortality associated with vaccine, Related to influenza pandemic of 1918, Historical abuse of Blacks, Guillain-Barre syndrome, Spiritual concerns) |
| Smith-Norowitz, et al.2021 [164] | Intention to take COVID-19 vaccine: gender, smoking status and age subgroups/reasons for hesitancy: efficacy of the vaccine; side-effects; safety (based on newness of vaccine); cost to consumer; distrust/skepticism; or lack of correct information |
| Thaker J and Subramanian A. 2021 [165] | misinformation and hesitancy exposure conditions/associated: Education; Ethnicity |
| Uvais, A.2021 [166] | intention to receive COVID-19 vaccination: education; religion |
| Willis, et al.2021 [167] | associated with COVID-19 hesitancy: Screen Hours per School Day (Watching TV) |
| Fernández-Penny, et al. 2021 [168] | determinants of vaccine hesitancy: Gender; Race; Medical insurance type; Age; Immunocompromised status; Hospital or clinic visit < 6 months; Additional members of household; Highest level of education/Attitudes and opinions affecting vaccine hesitancy: Confidence in vaccine science (Vaccines are founded on false science, Vaccines can cause detrimental health issues, The best way to beat COVID is by having most people getting the infection rather than getting vaccinated, Some batches of vaccines from the same manufacturer are safer than others, Vaccines are effective in preventing disease spread/ infection, Face masks work to slow the spread of COVID, The COVID-19 vaccine from one manufacturer might be safer or better than a COVID-19 vaccine from another manufacturer; Trusted health/vaccine information sources (Government communications, Traditional news, Friends/family, Online blogs/forums, Social media; Scenarios that would impact health decision (Your primary care physician or family doctor urges you to get vaccinated, Your children, spouse, or other immediate family encourage vaccination, A large study comes out showing no side effects in a large cohort of people you relate with, There is new scientific data that shows no long-term side effects, A number of your friends are vaccinated and do not experience any side effects, A celebrity you look up to is vaccinated without side effects |
| Chen, et al.2021 [169] | **Reasons Behind the Vaccine Hesitancy:** concerns about the safety and effectiveness of vaccines; demand for more information, their anti-vaccine attitudes/emotions/beliefs, and a lack of trust in vaccine development |
| Paris, et al.2021[170] | **associated with COVID-19 vaccine intention:** age; occupation; ﬂu vaccine history; and controversy over the AstraZeneca vaccine tolerability/determinants of vaccine hesitancy: communication and media environment; income; education level; occupations; inﬂuential leaders; religion or cultural issues; knowledge/awareness ratio; perception of risk-beneﬁt balance; role of healthcare providers; etc. |
| Ekstrand, et al.2021 [171] | female; lack of confidence in vaccine safety, concerns about side effects and efficacy, and distrust in many of the common sources of vaccine-related information |
| Longchamps, et al.2021 [172] | Factors associated with COVID-19 vaccine hesitancy: Sex; Household composition; no legal residence in France; health literacy |
| Waters, et al.2021 [173] | factors associated with COVID-19 vaccine hesitancy: Gender; Education |
| Saluja, et al. 2021 [174] | Race/ethnicity and income were independently associated with vaccine hesitancy; Reason: Worried about COVID-19 vaccine effects or safety; Don’t think the vaccine will work; Don’t trust the government to develop a COVID-19 vaccine; Don’t trust the pharmaceutical companies to develop a COVID-19 vaccine; Want to wait to see how it works first; Don’t believe in getting vaccinated in general; Don’t think I will get COVID-19 |
| Townsel, et al.2021 [175] | Reasons for vaccine concerns: safety and effectiveness of the vaccine/vaccination intent: Pregnancy Status; Role; Race/ethnicity |
| Vieira Rezende, et al.2021 [176] | **associated with VH:** concurrent malignancy, ﬁbromyalgia, hydroxychloroquine use, and recent corticosteroid pulse therapy; patients with systemic lupus erythematosus; those treated with hydroxychloroquine, belimumab, and pulse corticosteroid therapy; and those with comorbidities such as chronic kidney disease, cancer, and ﬁbromyalgia/Reasons for COVID-19 vaccine hesitancy: Short duration of COVID-19 vaccine trials; Fear of vaccine-related side eﬀects Fear of ﬂare of their underlying disease Eﬃcacy uncertainty in immunocompromised persons; Lack of medical recommend on for vaccination Low eﬃcacy of currently approved vaccines in Brazil; Lack of conﬁdence in public health authorizes Acquired immunity due to prior COVID-19 infection; Fear of crowding at vaccination centre |
| Fisher, et al.2021 [177] | **associated with VH:** preferring to be informed about the COVID-19 vaccine via a conversation with one’s doctor and to be vaccinated at a doctor’s ofﬁce |
| Stoler, et al. 2021 [178] | **positively associated with vaccine willingness:** age-squared, household income, education, self-reported stress, and trust in health institutions/ negatively associated with vaccine willingness: age, Black race, conservative political ideology, and conspiracy thinking |
| Purnell, et al.2021 [179] | **Reasons for non-hesitancy:** Protection for self/family/community; Work/school requirement; Invalid response/no response; Travel; Limit mask wearing; Health care professional/Reasons for hesitancy: Mistrust of the health care system; Concerns with FDA approval timeline/ concerned with speed/research of vaccine development; Lack of information; Conﬁdent with current state of health; Fear; Religious beliefs; Unsure; Previous adverse reactions to other vaccines; Community/family inﬂuence |
