# Supplementary material for: A scoping review of COVID-19 vaccine hesitancy: refusal rate, associated factors, and strategies to reduce
Source: Front Public Health. 2024 Oct 15;12:1382849. doi: 10.3389/fpubh.2024.1382849 (PMC11518786; doi:10.3389/fpubh.2024.1382849)
Supplement: Supplementary file 3 [file Table_3.DOC]

**A Scoping Review of COVID-19 Vaccine Hesitancy; Refusal Rate, Factor Associated and Strategies to Reduce or Overcome**

**Journal of Public Health**

**Online Resource 3** Intervention/strategies suggested by individual studies to overcome vaccine hesitancy

| Authors/Year | Interventions |
| --- | --- |
| Abou Leila, et al. 2021 [180] | Following the implementation of counseling and patient education/ Rectifying the system problems and upgrading the physicians’ skills and knowledge elicited the vaccine concerns from the hesitant patient and helped in conducting tailored counseling. |
| Acheampong, et al.2021 [5] | Preventive vaccination programmes are combined with an enhanced and coordinated public education campaign. |
| Adigwe, 2021 [6] | Population concerns about safety should be highlighted and comprehensively addressed during enlightenment campaigns. Also, developing engagement strategies that clearly outline that beneﬁts associated with COVID-19 vaccines outweigh the associated risks, can help better inform the populace and consequently improve their acceptance of the intervention./Since it is critical for government and policymakers to develop contextual strategies aimed at achieving optimal immunisation, this emergent evidence can help improve uptake and reduce hesitancy./government’s responsibility for funding the ﬁrst phase of the immunisation campaign |
| Aemro, et al. 2021 [7] | provide brief information to the HCWs who are the role models for the general population. |
| Alabdulla, et al. 2021 [8] | States and healthcare authorities need to recognize the massive trust deficit around the vaccine and use the popular media used by people to share credible and reliable information. |
| Aldakhil, et al. 2021 [9] | health authorities take advantage of the media to increase awareness in the community by promoting credible information about vaccines/health facilities deliver appropriate educational programmes on COVID-19/public health ofﬁcials, such as those at the Ministry of Health (MOH), should engage in routine childhood vaccination community campaigns on a regular basis and use World Immunization Week to inform and clarify how these vaccines have contributed significantly to controlling many life-threatening diseases |
| Alfieri, et al. 2021 [10] | Efforts to disseminate information regarding the COVID-19 vaccine should be culturally tailored and delivered through effective sources to decrease the COVID-19 illness burden in disproportionately affected groups. |
| Ali, M and Hossain, A. 2021 [11] | Evidence-based educational and policy-level interventions |
| Allington, et al. 2021 [13] | Strengthening positive attitudes to vaccination and reducing conspiracy suspicions with regards to the coronavirus may have a positive effect on vaccine uptake, especially among ethnic groups with heightened vaccine hesitancy. |
| Almaghaslah, et al.2021 | design of Vaccine literacy programs according to the level of health, scientiﬁc, and general literacy of the high-priority groups. |
| Al-Mulla, et al.2021 [16] | data and information about the COVID-19 vaccine become available |
| Amuzie, et al. 2021 [18] | policymakers and stakeholders in the federal and state ministries of health should focus mainly on health education campaigns |
| Andrade, 2021 [19] | policymakers must devise programs that provide special vaccination incentives to members of marginalized ethnic communities. policymakers must also begin to consider a change in the traditional approach to health promotion in Venezuela. |
| Andrade, 2021 [20] | authorities also need to work towards greater inclusion of traditionally marginalized ethnic groups, and expand educational efforts towards the population at large/public health authorities need to engage Protestant communities in order to promote greater acceptance of scientific thinking |
| Ashok et al. 2021 [22] | a targeted approach toward raising awareness about the scientific process and transparency in vaccine development and manufacturing and the importance of mass vaccination may reduce vaccine hesitancy among HCWs and the general population. |
| Badr, et al .2021 [23] | for unemployed and low-income individuals who are experiencing ﬁnancial hardship, vaccine campaigns may need to emphasize that the vaccine is free of charge. For individuals who are more complacent about getting vaccinated, appeals that are framed to increase perceived susceptibility and that emphasize altruistic motives may be effective. For individuals experiencing convenience issues, interventions should aim at eliminating structural barriers and strengthening positive attitudes toward vaccination [45]. Finally, for individuals who lack conﬁdence in the vaccine or government, interventions that seek re- build public trust through a more uniﬁed public health messaging strategy that is adopted across government, scientiﬁc, and healthcare communities may go a long way toward overcoming vaccine hesitancy. |
| Bagasra, et al. 2021 [24] | community-based initiatives and building of partnerships between the scientific community and local community stakeholders. |
| Baniak, et al.2021 [26] | partnering with professional organizations and associated scientiﬁc journals to support effective communication; the overwhelming acceptance of the COVID-19 vaccine may be used to inﬂuence other hesitant nursing staff to accept vaccination. |
| Blanchi, et al. 2021 [29] | building a system of mutual trust, giving detailed information |
| Bogart, et al. 2021 [30] | interventions that are developed by and in partnership with communities, following community-based participatory research principles/local community empowerment is needed, combined with both national leadership and antiracist policies, to bring awareness and action to overcome the root causes of mistrust in systemic racism |
| Botwe, et al. 2021 [31] | public health educational intervention from stakeholders (through e.g. specialised CPD, seminars, conferences, talk shows etc)/there should be more proof of the efficacy of the various vaccines/more information and evidence to be shared on the safety of the vaccines, particularly, among their race and tribes./safety education |
| Bou Hamdan, et al.2021 [32] | evidence-based communication campaigns promoting vaccine acceptability should be designed and disseminated. Building vaccination trust among university students through the spread of clear messages is key to the success of vaccinating many. |
| Caserotti, et al. 2021 [33] | perception of COVID-19-related risk |
| Chadwick et al. 2021 [34] | First, direct contact, through the post, workplace, or community structures, and through phone counseling via local health services, could reach the news avoiders./Second, TV public information advertisements should point to authoritative information sources, such as National Health Service (NHS) and other public health websites, which should then feature clear and simple ways for people to share material among their online social networks./ Third, informative social media campaigns will provide super seekers with good resources to share, while also encouraging the social media dependent to browse away from social media platforms and visit reliable and authoritative online sources./ Fourth, social media companies should expand and intensify their removal of vaccine disinformation and anti-vax accounts, and such efforts should be monitored by well-resourced, independent organizations. |
| Chaudhary et al. 2021 [35] | Strategies to raise awareness of the benefits of vaccination; Health professionals should work together with community leaders to provide accurate information and knowledge regarding vaccination to clarify the misconceptions relating to the risk and safety concerns. |
| Chen, et al. 2021 [169] | reinforce the publishing of information regarding the safety and validity of COVID-19 vaccines and incentives of vaccination completion/advocate for more volunteers to engage in motivating their friends or family members |
| Cordina, et al 2021 [38] | COVID-19 vaccination information campaigns should promote group strategies, focusing on emphasising the safety of the vaccine and offer reassurance, especially to women. |
| Danabal, et al. 2021 [40] | design effective behavior change communication campaigns |
| Dinga, et al.2021 [42] | community/public engagement |
| Dror, et al. 2020 [44] | Interventional educational campaigns targeted towards populations at risk of vaccine hesitancy are therefore urgently needed to combat misinformation and avoid low inoculation rates. |
| Du, et al. 2021 [45] | tailored public health measures are needed to increase perceived susceptibility and beneﬁt, and decrease perceived barriers among reproductive women. |
| Duong, et al.2021 [46] | health literacy intervention on the individual, interpersonal, and organizational levels |
| Edwards, et al. 2021 [47] | public health messaging; alternative policy measures |
| Ehde, et al. 2021 [48] | developed a COVID-19 Vaccine Conversation Tip Sheet for healthcare providers (It includes tips for eliciting and validating the hesitant patient’s perspective, addressing vaccine misconceptions, and developing a plan for future steps or discussions)/providing vaccinations at convenient places (e.g., clinics, churches, job sites) or scheduling default vaccine appointments |
| Freeman, et al. 2020 [52] | Vaccine public information that highlights prosocial benefits may be especially effective. |
| Freeman, et al. 2021 [53] | alternative needle- free delivery routes of COVID-19 vaccination could be made available. /administration of the vaccine by injection could be made more tolerable, producing positive narratives in the general public of the process./there could be wider provision of the brief psychological treatments (exposure therapy and applied tension) for blood-injection-injury fears that show large treatment effects |
| Gao, et al. 2021 [56] | establish a standardized COVID-19 vaccine course, and provide vaccination services on campus. |
| Gaur, et al. 2021 [57] | educating AIRD patients regarding the pros and cons of vaccination, particularly concerning immunological disease; The message should clearly penetrate that there is a negligible risk of AIRD-flares with the COVID-19 immunization and the side effects are mild and manageable. |
| Geana, et al. 2021 [58] | health education, as well as mitigation for mistrust, misinformation and conspiracy theories |
| Gehlbach, et al. 2021 [59] | interventions that decrease structural vulnerabilities by addressing issues of (dis)trust in government and public health among this population. |
| Gerussi, et al. 2021 [61] | educational interventions |
| Griffith, et al. 2021 [62] | introduce campaigns that educate the public about using clear language in media that are commonly used to digest content (eg, social media); Have nonpolitical, respected older adult Canadian celebrities take the vaccine as an example; Such celebrities could be retired athletes or musicians; Emphasize that vaccines are rooted in science and not politics. This is a difficult quality to understand; In action, this could be done by having messages come from trusted physicians instead of politicians; Highlight examples of instances when the vaccine has worked; Reiterate the safety of the vaccine; Reiterate the fact that the steps in the scientific development of the vaccine were not missed. |
| Harrison, et al. 2021 [63] | develop provider-academic-community partnerships |
| Holeva, et al. 2021 [65] | build solid communication channels minimizing the uncertainty and preventing prolonged unnecessary avoidance behaviors. /ongoing monitoring |
| Hou, et al. 2021 [67] | develop an effective vaccine campaign |
| Hwang, et al. 2021 [68] | first strategy should be to address the need to enhance effective communication by providing evidence of the vaccine’s efficacy, thereby resolving the misunderstanding among the people. The epidemiologic and socioeconomic factors associated with a high risk of vaccine hesitancy should be considered for targeted communication. |
| İkiışık, et al. 2021 [69] | building trust to support the public acceptance of a potential COVID-19 vaccine |
| Jain, et al. 2021 [70] | Heightened risk perception regarding COVID-19; health education programmes tailored to boost awareness regarding vaccine and improve trust in government agencies/Focus should be on promoting official sources of information to counter apprehension generated through social media use. |
| Jin, et al.2021 [72] | the role of communication messages and medium selection to support vaccination campaigns; fear appraisal messages persuading people to get early vaccines; the usage of public service messages incorporating such fear appraisals through traditional media |
| Khaled, et al.2021 [74] | public education |
| Khan, et al. 2021 [75] | communication strategies to reduce vaccine hesitancy should be tailored to younger populations of speciﬁc age groups rather than reliance on a one-size-ﬁts-all communication strategy. |
| King, et al. 2021 [76] | Messaging about safety, addressing trust, and clarifying the value of vaccinations to prevent COVID-19 is needed. |
| Knight, et al. 2021 [77] | development of a scalable digital intervention which seeks to address the concerns of individuals who are vaccine hesitant with a view to enhancing their confidence in COVID-19 vaccines and, in turn their uptake. |
| Kobayashi, et al. 2021 [181] | maintaining optimistic messaging about vaccine development may be an effective communication strategy to minimize the bias around the globe that prior studies found as being potential hurdles to Covid-19 vaccine acceptance. |
| Kose, et al. 2021 [78] | information programmes can be organized for older healthcare professionals. |
| Kumar, et al. 2021 [79] | Education about the vaccine’s safety and eﬃcacy |
| Lamot, et al. 2020 [81] | centrist political parties and their leaders could also have a posi- tive impact on their voters and supporters if they communicate the personal and public health significance of the uptake of a safe and efficient Covid-19 vaccine. / it is essential that healthcare institutions, physicians, nurses and other healthcare professionals, in communication with patients and the general population, emphasize that the vaccination protects not only ourselves but also other individuals./the public, institutions and decision-makers have common goals and only alongside evidence-based public health campaigns. |
| Li, et al. 2021 [82] | Physician-led engagement; providing accurate information through reliable sources; Offering an open, honest, and potentially anonymous dialogue where individuals discuss their concerns with health care professionals |
| Liu, et al. 2021 [83] | The government and different media platforms should encourage the dissemination of correct information about vaccines, the communities, and medical staff to improve residents’ knowledge about vaccines and strive to improve residents’ electronic health literacy. government provided timely information (such as side effects of the vaccine) by social media, and adequate protective supplies might mitigate the level of the hesitation of the COVID-19 vaccine. |
| Liu, and Li, 2021 [84] | using multiple languages in various forms of communications to dispel misinformation and promote vaccination, developing outreach programs featuring community leaders and local health care providers whom the community members find more trustworthy and relatable, creating more flexible and accessible appointment systems to remove technological barriers, organizing virtual town hall meetings and dialogues alike to directly address people’s vaccine-related questions and doubts, and so on. |
| Lockyer, et al. 2021 [86] | there has to be systematic monitoring of the circulation of misinformation on social media. /they did trust people within community support roles that they had frequent contact with, for example teachers, nursery workers and advice workers. Effectively harnessing these connections, through trusted community networks and providing information in languages spoken locally, will be central to ensuring the spread of correct information and providing reassurance./It would be prudent for health, social and community workers to be provided with an updated summary of locally circulating misinformation with helpful resources to help them counter concerns and provide informed reassurance. |
| Lucia, et al. 2021 [87] | educational curriculum designed to enhance student knowledge about the COVID-19 vaccine and to teach vaccine counseling skills./medical students who are vaccinated have positive attitudes towards vaccines, and it is hoped they will be able to share their vaccination experiences with their patients and encourage vaccine uptake. |
| Mahdi, 2021 [88] | medical students as leaders and guides in the health system for increased planning and education of the population for COVID-19 vaccine acceptability. |
| Mangla, et al. 2021 [89] | Understanding public trust and conﬁdence during health emergencies is vital to enhance trust and promote conﬁdence and compliance with recommendations and measures. Understanding the threat posed by anti-vaccination efforts on social media and the impact they have on individual attitudes is also critically important to ensure the success of worldwide COVID- 19 vaccination programs./guiding public health program development, supporting the education needs of individual communities, addressing misinformation, and guiding global practice change. |
| Maraqa, et al. 2021 [90] | create interventions to alleviate the fear and misunderstandings about the COVID vaccines among health professionals |
| McElfish, et al. 2021 [91] | Vaccination programs and interventions must consider these differences in COVID-19 vaccine hesitancy and general vaccine trust to alleviate COVID-19 disparities. |
| Mejri, et al. 2021 [92] | Cancer patient’s education about the impact of the vaccine on their disease and on the COVID-19 is needed. |
| Mollalo, and Tatar, 2021 [94] | engaging community groups, champions, and faith leaders/wider communication through media and fighting against widespread misinformation would raise public awareness about the scale and consequences of the COVID-19 pandemic. |
| Momplaisir, et al. 2021 [95] | Developing messaging emphasizing the individual, family, and community benefits of getting the vaccine and providing continued transparency on the safety profile of COVID-19 vaccines |
| Muhajarine, Net al. 2021 [99] | Targeted and accurate messaging to specific socio-demographic groups who are less likely to be vaccinated, and encouragement and modeling by people who they trust/ensuring a successful vaccination campaign will also entail rebuilding public trust through transparent action, clear communication and demonstrated accountability of the key stakeholders in our society, including governments and health care systems. |
| Murphy, et al.2021 [101] | need for public health ofﬁcials to disseminate information via multiple media channels |
| Musa, et al.2021 [102] | Effective campaigns of health promotion awareness; Effective communication strategies |
| Navarre, et al. 2021 [103] | Targeted written and oral information campaigns will be necessary to improve vaccination coverage/Messages should be designed to increase conﬁdence in the safety of vaccination, to correct misconceptions, to raise knowledge and awareness, and to overcome mistrust in authorities. All information channels should be used to deliver these messages, including social networks. |
| Nazlı et al. 2021 [104] | ways to spread accurate information about the vaccine in a healthier way |
| Nguyen, 2021 [105] | Developing messaging emphasizing the individual, family, and community benefits of getting the vaccine and providing continued transparency on the safety profile of COVID-19 vaccines. |
| Okoro, et al. 2021 [106] | Collaborating with communities to host vaccine clinics in community spaces and employing community members as outreach coordinators and navigators greatly eliminates accessibility-related issues. |
| Oliveira, et al. 2021 [108] | authorities must prepare the population with more effective messages, aligning the political, religious, and health discourse around the benefits of immunization. |
| Park, et al. 2021 [110] | the government needs to put more effort into communication with those who are liberal or “no political opinion”, younger, and have low levels of government trust. policy development is required to consider additional political tendencies, government trust, etc. |
| Reno, et al. 2021 [115] | Social media; transparency and widespread diffusion of information on the processes behind vaccine approval and distribution; institutional websites; Institutions and public health organizations could land on even more social media, with official profiles embracing a style of communication in line to the different social media and through advertisement. |
| Reno, et al.2021 [116] | inform policy makers and public health professionals in their efforts to target information and communication campaigns to counteract vaccine hesitancy. |
| Riad, et al. 2021 [117] | designing educational programs |
| Riad, et al. 2021 [118] | further implementation of infectious diseases epidemiology education and vaccination trends within undergraduate dental curricula. |
| Robertson, et al. 2021 [119] | the subgroups we have identified as being vaccine hesitant should be included in the planning and development of any engagement programmes./ by working in close partnership with communities and making use of community champions./universal and targeted educational interventions /Full endorsement from regulatory bodies/ efforts to combat misinformation, especially around vaccine safety/ A concerted effort to engage with younger adults both online and through traditional communication channels |
| Rozek, et al. 2021 [121] | Health professionals should, in turn, enlist religious leaders when developing and deploying their communication strategies./Public trust in science and conﬁdence in the health ministry |
| Sadaqat, et al. 2021 [122] | Multidisciplinary education, pro-vaccination awareness campaigns, and health seminars must be introduced by government authorities to promote a positive attitude towards vaccines. |
| Saied, et al. 2021 [123] | Provision of evidence‐based information for COIVID‐19 vaccines with effective and proactive initiatives that keep a vigilant eye to fight misinformation./Organizing expert groups from health professionals and scientists for scientific engagement on COVID‐19 vaccines to provide truthful and understandable information for reducing confusion and doubt and reconstructing a trusting relationship with the public utilizing social and traditional media./Monitoring and confronting misinformation and fake news on COVID‐19 Vaccines, especially on the social media platforms. |
| Silva, et al.2021 [129] | knowing the audience and their needs/Targeting education on vaccine-preventable diseases, immunology, and critical thinking based on student’s level of education, delivered through multiple communication channels/Understanding the spectrum of vaccine hesitancy |
| Sowa, et al.2021 [131] | Knowledge, i.e., the result of research focused on the questionable issues (in public perception) should be shared with the general population in a comprehensive and personalized manner (according to the educational attainment). The recommendation system should focus on health care professionals in accordance with the principles of Evidence Based Medicine./Effective campaigns promoting vaccination should be considered on the decentralized, local level |
| Talmy, et al.2021 [182] | On-site COVID-19 vaccine rollout joined with primary care communication interventions may maximize vaccine uptake within a young-adult community. |
| Thaker, 2021 [132] | health communication campaign from trusted sources, with information that addresses prevailing concerns about vaccines, is likely to help increase COVID 19 vaccine uptake. |
| Tram, et al. 2021 [135] | public health and medicine must be aligned with the social realities of America experienced by different populations; health systems and government entities must also accept responsibility for the present-day stunted public engagement with public health. Segmented solutions to reach into sequestered social systems are needed to optimize vaccine uptake, but longer-term institutional building is needed to win trust and rebuild the social contract. |
| Tsai, et al. 2021 [137] | Healthcare related social media forums which rapidly disseminate accurate information about the COVID-19 vaccine may play an important role. |
| Turhan, et al.2021 [138] | health communication practitioners and health system authorities need to recognize the mechanism of health care system distrust, health literacy, and vaccine hesitancy. |
| Uzochukwu et al. 2021 [141] | designed advocacy and behavior-change communication messages that target the respective segments of university community members |
| Vallée, et al. 2021 [142] | communication strategy, emphasizing the collective benefits of herd immunity in the population living with HIV and reassuring patients with the chronic disease about the safety of the proposed vaccines. |
| Vergiev, S. and Niyazi, D. 2021 [143] | need of information campaigns and other additional actions that will raise public awareness and knowledge about the positive effects of the vaccines |
| Wang, et al. 2021 [145] | provide accurate and timely education on the COVID-19 vaccine and adopt comprehensive measures to improve the population’s willingness and confidence before vaccination |
| Wang, et al. 2021 [146] | start promotion, initiate policy-making and set up priority guidelines for the vaccination before the vaccines are approved |
| Wang, et al. 2021 [147] | increased communication on the benefits and safety of vaccines; encouraging conversation among local community members, neighbors, co-workers, friends, and family. /strategies to combat hesitancy would greatly benefit from collaborations with trusted community partners (academic, healthcare providers, and community agencies) and the implementation of public health initiatives./Key elements to reduce vaccine hesitancy should be practical and aimed toward minority ethnic groups with low vaccine uptake to reduce misinformation and increase confidence. |
| West, et al. 2021 [149] | On a global scale, efforts such as the Sustainable Development Goals (SDGs) high- light the interconnectedness of systems and the importance of public participation to ensure health systems can achieve their mission of improving the health of populations. Applying these principles and those of the World Health Organization’s program on Financing Common Goods for Health can help structure the multisectoral response needed to leverage the relatively high vaccine acceptance of TFWs and address the structural vulnerabilities that lead to hesitancy in this population. |
| Willis, et al. 2021 [150] | public health messaging for the COVID-19 vaccine must consider the role of people’s fears of infection, general trust in vaccines, and the historical and ongoing mistreatment of many racial/ethnic minorities. |
| Wong, et al.2021 [151] | facilitation measures to acquire vaccination among unvaccinated subjects: Granting reasonable travel expense allowance; Granting leaves on the day of vaccination and the day after; Vaccine passports for overseas travel; Relaxing restrictions on religious activities; Relaxing restrictions on visiting policies in hospitals and healthcare facilities; Relaxing mandatory isolation; Resumption of face–to–face teaching in schools; Allowance to enter entertainment venues; Recommendation by doctors; Recommendation by family members or relatives; Recommendation by colleagues and friends; Recommendation by employers; Recommendation by the government/the government should strengthen public education and information dissemination to tackle vaccine hesitancy. The government should proactively provide transparent and comprehensive information of the vaccination, work with physicians to eliminate any public misconception, and make use of multiple incentives measures. |
| Xu, et al. 2021 [152] | strengthening knowledge regarding the safety and importance of the vaccine |
| Xu, et al. 2021 [153] | Health authorities and medical specialist teams should strengthen the dissemination of vaccination-related knowledge for patients such as an expert consensus or guidelines through various media. Some key points should be emphasized in the knowledge about vaccination, such as the signiﬁcance of vaccination, the safety and side eﬀects of COVID-19 vaccination and predicting of epidemiological trends of COVID-19. |
| Zhuang, et al. 2021 [157] | Evidence-based patient education |
| Barello, et al. 2020 [183] | Understanding the student’s perspective about the future COVID-19 vaccine and supporting their health engagement and consciousness may be useful in planning adequate response and multidisciplinary educational strategies—including the psychological perspective on vaccine hesitancy underlying factors - in the post-pandemic period. |
| Bass, et al. 2021 [159] | Immediate planning for how best to communicate about the beneﬁts and address concerns about perceived risks of vaccination for these at-risk groups will be a way to ensure that negative health eﬀects of COVID-19 are mitigated. |
| Carcelen et al.2021 [160] | providing accurate information through trusted sources of information/National vaccine deployment plans should include community sensitization tailored for the adult population. |
| Ebrahimi, et al. 2021 [161] | transparency in policy- making decisions regarding the vaccination program and clear provision of information about the rigorous process that underlie the approval of new vaccines. the involvement and aid of other community leaders |
| Khubchandani, et al.2021 [162] | variety of messaging strategies |
| Berry, et al.2021 [163] | Sharing positive emotions and stories |
| Smith-Norowitz, et al.2021 [164] | implementation of effective education |
| Thaker J and Subramanian A. 2021 [165] | Misinformation correction campaigns should not only focus on outright misinformation but also on vaccine hesitancy, such as side effects, the need for vaccination across age groups, and clarifying the extremely rare risks associated with vaccines. Public health ofﬁcials should not hesitate to transparently inform people of the limitations of vaccinations either, providing them with an authoritative resource rather than driving them to alternative sources that may misinform the public./Messages addressing individuals’ concerns, through messengers they trust, are likely to help overcome vaccine hesitancy and improve COVID-19 vaccination rates |
| Uvais, A.2021 [166] | tailored public health interventions targeting vaccine hesitancy among this vulnerable population should be prioritized |
| Chen, et al.2021 [36] | first, governments should issue consistent recommendations, and establish a reliable health publicity system in order to improve public acceptance of the COVID-19 vaccine. Second, targeted interventions are necessary to improve the coverage of vaccination. For example, staff members of institutions could take the lead in vaccination to play an exemplary role. Third, through effective consultations, medical staff are increasing the coronavirus vaccine’s awareness and acceptance. |
| Ekstrand, et al.2021 [171] | Campaigns may benefit from using trusted sources, including antiretroviral therapy center staff, providing clear information about safety and efficacy and emphasizing the role of vaccines in preventing severe disease, hospitalizations and death, and the reduction of forward transmission to unvaccinated household members. |
| Waters, et al.2021 [173] | oncology provider recommendations |
| Saluja, et al. 2021 [174] | Public health departments and health systems must collaborate with community-based organizations to develop culturally appropriate messaging to lower-income and racial/ethnic communities./develop effective alternatives to online vaccine appointments and utilize non-web-based platforms to disseminate in- formation about the safety and efficacy of the vaccine |
| Vieira Rezende, et al.2021 [176] | Attending physicians should spend more time with IMID patients who bear risk characteristics for COVID-19 vaccination, reinforcing the overall safety and efﬁcacy of COVID-19 vaccines, including among immunocompromised individuals, so that we can achieve global mass vaccination. |
| Fisher, et al.2021 [184] | Incorporating healthcare providers in the COVID-19 vaccination process |
| Stoler, et al. 2021 [178] | Difficult community dialogues may be a crucial first step toward engaging communities of color and promoting COVID-19 vaccine acceptance. |
| Purnell, et al.2021 [179] | guidelines and strategies: audience targeting, barrier analysis, community engagement, marketing and promotion, outreach, and digital media/education/encouragement from a trusted individual/determine individual concerns and respond to them in a culturally competent manner, rather than arguing with patients about vaccination/Working with community organizations to reach out and empower patients through education and resources/Successful models of community-based approaches for health-related outreach in minority communities/Creating educational and training programs focused on addressing social determinants of health, racial bias, and stigma affecting health care/ use of digital platforms to provide targeted messaging about the COVID-19 vaccine/Messaging aimed at correcting misinformation on Instagram, YouTube, and TikTok and promoting vaccine confidence |
